# Developing a toolkit to implement the Statin Choice Conversation Aid at scale: application of a work reduction model

**DOI:** 10.1186/s12913-019-4055-8

**Published:** 2019-04-24

**Authors:** Aaron L. Leppin, Kasey R. Boehmer, Megan E. Branda, Nilay D. Shah, Ian Hargraves, Sara Dick, Glyn Elwyn, Henry H. Ting, Siqin Ye, Ryan Gilles, Marghoob Abbas, Alex Alexander, Victor M. Montori

**Affiliations:** 1 Division of Health Care Policy and Research, Mayo Clinic, 200 First Street SW, Rochester, MN 55905 USA; 2 Knowledge and Evaluation Research Unit, Mayo Clinic, 200 First Street SW, Rochester, MN 55905 USA; 3 Department of Health Sciences Research, Mayo Clinic, 200 First Street SW, Rochester, MN 55905 USA; 4 The Dartmouth Institute for Health Policy and Clinical Practice, Level 5, Williamson Translational Research Building, 1 Medical Center Drive, Labanon, NH 03756 USA; 5 Department of Cardiovascular Diseases, Mayo Clinic, 4500 San Pablo Rd S, Jacksonville, FL 32224 USA; 6 Department of Medicine, Columbia University Medical Center, 622 West 168th Street, New York, NY 10033 USA; 7 Department of Family Medicine, Kootenai Health, 2003 Kootenai Health Way, Coeur d’Alene, ID 83814 USA; 8 Mosaic Life Care, 5325 Faraon Street, St. Joseph, MO 64506 USA

**Keywords:** Shared decision making, Implementation, Scale-up, Spread, Implementation toolkit, Statins, Statin choice decision aid, Statin choice conversation aid, Implementation strategies

## Abstract

**Background:**

Guidelines recommend shared decision making (SDM) for determining whether to use statins to prevent cardiovascular events in at-risk patients. We sought to develop a toolkit to facilitate the cross-organizational spread and scale of a SDM intervention called the Statin Choice Conversation Aid (SCCA) by (i) assessing the work stakeholders must do to implement the tool; and (ii) orienting the resulting toolkit’s components to communicate and mitigate this work.

**Methods:**

We conducted multi-level and mixed methods (survey, interview, observation, focus group) characterizations of the contexts of 3 health systems (*n* = 86, 84, and 26 primary care clinicians) as they pertained to the impending implementation of the SCCA. We merged the data within implementation outcome domains of feasibility, appropriateness, and acceptability. Using Normalization Process Theory, we then characterized and categorized the work stakeholders did to implement the tool. We used clinician surveys and IP address-based tracking to calculate SCCA usage over time and judged how stakeholder effort was allocated to influence outcomes at 6 and 18 months. After assessing the types and impact of the work, we developed a multi-component toolkit.

**Results:**

At baseline, the three contexts differed regarding feasibility, acceptability, and appropriateness of implementation. The work of adopting the tool was allocated across many strategies in complex and interdependent ways to optimize these domains. The two systems that allocated the work strategically had higher uptake (5.2 and 2.9 vs. 1.1 uses per clinician per month at 6 months; 3.8 and 2.1 vs. 0.4 at 18 months, respectively) than the system that did not. The resulting toolkit included context self-assessments intended to guide stakeholders in considering the early work of SCCA implementation; and webinars, EMR integration guides, video demonstrations, and an implementation team manual aimed at supporting this work.

**Conclusions:**

We developed a multi-component toolkit for facilitating the scale-up and spread of a tool to promote SDM across clinical settings. The theory-based approach we employed aimed to distinguish systems primed for adoption and support the work they must do to achieve implementation. Our approach may have value in orienting the development of multi-component toolkits and other strategies aimed at facilitating the efficient scale up of interventions.

**Trial registration:**

ClinicalTrials.gov NCT02375815.

**Electronic supplementary material:**

The online version of this article (10.1186/s12913-019-4055-8) contains supplementary material, which is available to authorized users.

## Background

### The statin choice conversation aid

Statins are medications used for the primary prevention of cardiovascular disease [1, 2]. The Statin Choice Conversation Aid (SCCA) was developed through principles of user-centered design [3] to promote shared decision making (SDM) between patients and clinicians considering statins. The SCCA was tested in several randomized trials [4–7] and most of the results suggested the conversation aid led to increased patient knowledge, greater patient comfort with the decision making process, and better alignment of prescription with estimated cardiovascular risk; one trial showed improvement in patient self-reported adherence to treatment at 3 months [4]. Subsequently, the SCCA was integrated into the Mayo Clinic electronic medical record (EMR) and made available for passive external dissemination on the Mayo Clinic Shared Decision Making National Resource Center website (statindecisionaid.mayoclinic.org). This passive dissemination had resulted in more than 166,000 hits on the website in 2016, but use of the tool from within the EMR remained limited to Mayo Clinic [8]; limited evidence of adoption by Mayo and other organizations existed [9].

In 2013, the American College of Cardiology/American Heart Association (ACC/AHA) guidelines for cholesterol management recommended that decisions to initiate statin therapy be based on calculations of individualized risk and incorporate patient values and preferences through a SDM approach [1, 2]. The ACC/AHA specifically promoted use of the SCCA in its performance measures related to cardiovascular prevention [10]. Further, the Centers for Medicare and Medicaid Services, in partnership with the Million Hearts initiative, started a demonstration project focused on using SDM for cardiovascular risk reduction [11]. Together, this resulted in increased interest nationally in the routine use of SDM and other tools to support the decision to initiate statins for primary prevention.

### Scale-up, spread, and the role of toolkits

In such situations where the desire to implement an intervention is widespread across organizations, broadly applicable and efficient strategies for facilitating cross-organizational scale-up and spread are of potential value [12–15]. Yet, most research in this area has been conceptual and few successful examples of such strategies exist in clinical settings [15]. Indeed, a 2016 review of models and frameworks for scale-up and spread suggested that most scale-up efforts in clinical settings are based on quality improvement (QI) methods intended only to scale practices within a single organization [16]. The few examples of effective strategies aimed at replicating best practices and improving processes across multiple organizations (such as the Institute for Healthcare Improvement (IHI)‘s “Breakthrough Series,” or BTS, model) often include external facilitation, co-learning, and a significant investment of time and resources from committed organizations. More efficient, low-touch, and so-called “non-sequential” or “campaign-based” approaches [16, 17] for facilitating the spread of interventions are appealing because they have the potential to reach a greater and more diverse set of organizations. Implementation “toolkits” are examples of such approaches, although they have been conceptualized in varying ways in the literature and in practice [18–22]. For the purposes of this paper, toolkits comprise a heterogeneous bundle of evidence-based strategies designed to be disseminated to and taken up and used (as a multi-faceted implementation strategy [23]) by implementing stakeholders in distant organizations. Unfortunately, and despite their theoretical value, implementation toolkits—as typically designed and disseminated—have not proven consistently effective at achieving implementation, particularly when compared to more “hands-on” multi-faceted strategies [24–26]. One likely reason for this is that, in many cases, toolkits are not designed to target specific and known barriers to implementation or to support stakeholders in carrying out the work most critical for success. This is supported, for example, by studies showing that the number of components of many multi-faceted implementation strategies do not correlate with effectiveness and the suggestion that a “kitchen sink” approach is frequently taken [24, 27]. Developing toolkits and other multi-faceted strategies in this sort of haphazard way may result in the inclusion of strategies and components that are of low value and that only serve to increase stakeholder work and confusion. Partly for these reasons, Barker and colleagues, in their 2016 Framework for Going to Full Scale, suggested that toolkit-like strategies only be deployed after “learning deeply from a small number of sites” and developing the “scalable unit” [16]. This line of thinking is also consistent with a much larger body of literature and understanding highlighting the importance of incorporating theory, experience, and data in the design of implementation strategies [25, 28, 29].

### The AIDED model for scale-up

The AIDED (Assess, Innovate, Develop, Engage, and Devolve) Model for Scale-Up [30] requires implementers to first carefully assess the implementation process in a purposeful sample of index contexts. During this assessment, they must take note of the opportunities for intervention modification and innovation and the pre-existing communication channels that can facilitate spread. The AIDED model likely has heuristic value in organizing and prescribing the activities of scale-up efforts in and across clinical settings, although its individual phases lack a robust theoretical basis for informing the development of implementation strategies, such as toolkits.

### Objective

We sought to use the AIDED model—supplemented with phase-specific theoretical guidance—to develop a theory-based toolkit for facilitating implementation of the SCCA in diverse and routine clinical settings with minimal outside intervention. In this paper, we describe the experience of and insights gained from implementing the SCCA within three healthcare organizations and explain how these were used to develop the resulting toolkit.

## Methods

### Conceptual rationale and study overview

In this project, we sought to characterize the work stakeholders in multiple organizations needed to do to implement the SCCA and to develop a toolkit that could mitigate or ease this work. These activities corresponded to the Assess and Innovate phases of AIDED and included elements of formative evaluation [31] and action research [32], respectively. In the AIDED phases of Develop and Engage we sought to identify and partner with the communication channels and pre-existing networks that could “Devolve” the strategy.

During the Assess phase we first characterized the baseline contexts (through application of the Consolidated Framework of Implementation Research [33]) of each participating organization according to their perceived effects on the feasibility, acceptability, and appropriateness of implementing the SCCA. We took this approach because we hypothesized that much of the initial work that an organization would need to do to improve the potential and capacity for implementation [34, 35] would involve changing the intervention and environment to optimize these outcome domains [35–37].

**Fig. 1:**
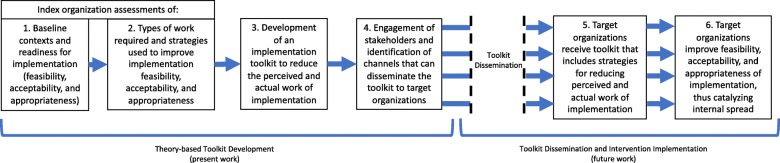
Study flow diagram (steps 5 and 6 are hypothetical and were not the focus of this work)

**Fig. 2:**
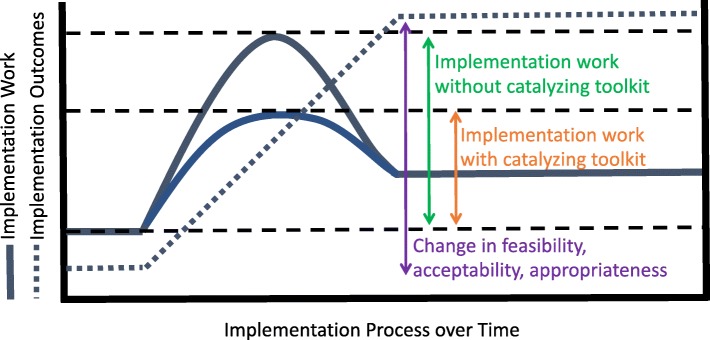
Conceptual rationale

We then used Normalization Process Theory (NPT) [38]—which considers and categorizes the work of implementation into constructs of coherence, cognitive participation, collective action, and reflexive monitoring (see Table 1)—to guide the formative evaluation [31] of the implementation process. We paid careful attention to the types of work required, the emergent strategies [39, 40] organizations used to do this work, and how these were allocated to optimize the feasibility, acceptability, and appropriateness of implementation in each context. Finally, we employed user-centered design and stakeholder engagement strategies that we have used in other projects [3] to translate this insight and to guide activities in the Innovate, Develop, and Engage phases. We likened the entire project to the development of a catalyst that could be disseminated to facilitate widespread implementation of the SCCA. We drew the metaphor of a catalyst from biochemistry, wherein enzymatic catalysts are used to reduce the energy threshold (e.g. the contribution of work) required for a chain reaction to occur. Indeed, in biochemistry, when catalysts are present, energy thresholds are reduced and reactions happen more readily. Extending this logic to implementation, we hypothesize that when implementation work is reduced, implementation will happen more readily. An overview of the study activities and purpose is presented in Fig. 1. The biochemistry-based conceptual rationale is presented in Fig. 2. Specifically, Fig. 2 shows how implementation work must increase significantly during initial implementation and remain elevated—albeit to a lesser extent—to sustain the intervention. It also shows how a well-designed toolkit could function to reduce the extent to which work must increase during initial implementation. A summary of key implementation work and outcome constructs is presented in Table 1.

**Table 1:** Key implementation work and outcome constructs

Construct	Definition
Implementation Work	
Coherence	The work agents (stakeholders) do to attribute meaning to an intervention and make sense of its potential within their context and their role in relation to it
Cognitive Participation	The work agents (stakeholders) do to legitimize and enroll (engage) themselves and others into an intervention and frame how participants become members of the related community of practice
Collective Action	The work agents (stakeholders) do to mobilize skills and resources and enact the intervention and frame how participants realize and perform the intervention in practice.
Reflexive Monitoring	The work agents (stakeholders) do to assemble and appraise information about the effects of the intervention and utilize that knowledge to reconfigure social relations and actions.
Implementation Outcomes	
Feasibility	The extent to which a new treatment, or an innovation, can be successfully used or carried out within a given agency or setting
Acceptability	The perception among implementation stakeholders that a given treatment, service, practice, or innovation is agreeable, palatable, or satisfactory
Appropriateness	The perceived fit, relevance, or compatibility of the innovation for a given practice setting, provider, or consumer; and/or perceived fit of the innovation to address a particular problem

### Participants and setting

In the Fall of 2014, we partnered with 3 healthcare organizations that were interested in implementing the SCCA. Eligible systems were autonomous and independent members of a pre-existing network affiliated with Mayo Clinic called the Mayo Clinic Care Network. Systems were classified by the vendor of their electronic health record and then selected at random for invitation to the study. The health systems served mostly white (> 90%), rural and suburban populations of all socioeconomic and educational backgrounds in the West, Upper Midwest, and Lower Midwest of the United States. Two of the systems (Systems 1 and 2) were integrated delivery systems spread over a larger geographic area. They employed 86 and 84 primary care clinicians (physicians, nurse practitioners, physician assistants), respectively. The third system (System 3) was smaller and localized to a single community. It comprised 26 primary care clinicians and included a family medicine residency program. Staff clinicians in all systems were fully dedicated to clinical care, with the majority of clinicians (84, 64.3, 76.9%) being physicians (in Systems 1, 2, and 3, respectively). To participate, all systems agreed to partnering with our research team to implement (and electronically integrate into the medical record) the SCCA across all of primary care and to forming a 5–10 member implementation team comprised of a physician lead and local experts in quality improvement and information technology. Patients, primary care clinicians, implementation team members, and health system leaders within each system were considered participants and were consented for all study activities.

### Measures and outcomes

We developed and piloted all measures for this study in the Summer of 2014. We designed the measures, to the extent feasible, to describe the work of the implementation process [38] and/or assess its outcomes [36, 41] as part of a multi-level organizational evaluation of SCCA implementation.

We created surveys for all parties (patients, clinicians, health system leadership, and implementation team) and collected them at 3 time points (baseline, 6 and 18 months). Patients were surveyed post-encounter with a measure [42] to assess their perceptions of the level of involvement in decisions generally. The patients were a convenience sample approached during a 4–8 week time period in which we requested at least 25 patient surveys per clinician at each of the 3 time points of data collection. This assessment was not connected to use of the SCCA but instead was intended to get a pulse of the organization and establish patient demographics. Clinicians were surveyed to ascertain their beliefs and attitudes toward SDM and the SCCA. Clinicians were also asked to report on whether they had been exposed to the tool and, if so, the frequency with which they were using it. Implementation team and leadership surveys were similar to clinician surveys, but were more focused on understanding respondents’ perceived readiness for the work of implementation and its relative priority, respectively.

For the qualitative data collection, we developed semi-structured interview guides for clinicians, implementation team members, and system leadership, as well as focus group guides for the implementation team. The qualitative assessment tools were intended to help us understand the effort, opportunity costs, and value propositions that were likely to either promote or inhibit implementation (conceptualized as the readiness for implementation) and the types and amount of work required to succeed.

Finally, we developed Google Analytics report procedures that would allow us to track the frequency of access to the SCCA based on organizational IP address and adopted a SCCA fidelity checklist that our team had used in prior projects [43] to assess fidelity. The Institutional Review Board of Mayo Clinic approved all measures and procedures for the study and the study was registered on ClinicalTrials.gov (Trial Number: NCT02375815). Copies of all measures are available in Additional file 1 and a summary of the measures—along with the outcomes they contributed to—are presented in Table 2.

**Table 2:** Summary of study measures and outcomes

Summary of Study Measures and Outcomes			
Assessment Tool	Measure	Outcome Domain	Collection Point
Implementation Context and Readiness Assessments			
Patient Survey	Involvement in decisions generally	Appropriateness	Baseline
Clinician survey	SDM beliefs scale	Appropriateness	Baseline
Clinician survey	SCCA beliefs scale	Acceptability	Baseline
Clinician survey	Self-report exposure and usage	Penetration	Baseline
Implementation team survey	Confidence that the strategy will succeed	Feasibility	Baseline
Leadership survey	Implementing SCCA is a priority	Feasibility	Baseline
On site system observations	Field notes, artifacts	Appropriateness	Baseline
Multi-level, Semi-structured Interviews	Inner and Outer Setting and Individual Characteristics	Acceptability, Appropriateness, Feasibility	Baseline
Implementation Work Assessment (post = 6 months)			
Multi-level, Semi-structured interviews	Implementation Process Evaluation	Allocation of Coherence, Cognitive Participation, Collective Action, Reflexive Monitoring work	Post
Implementation Team Focus Group	Implementation Process Evaluation	Allocation of Coherence, Cognitive Participation, Collective Action, Reflexive Monitoring work	Post
Implementation Outcome Assessment (post = 6 months; follow-up = 18 months)			
Patient Survey	Involvement in decisions generally	Appropriateness (change)	Post, follow-up
Clinician survey	SDM beliefs scale	Appropriateness (change)	Post, follow-up
Clinician survey	SCCA beliefs scale	Acceptability (change)	Post, follow-up
Clinician survey	Self-report exposure and usage	Penetration (change)	Post, follow-up
Google Analytics	System SCCA usage	Penetration, Sustainability (change)	Monthly throughout study
Standardized encounter observation	Fidelity checklist	Fidelity	Post

### Procedure

#### Assess

##### Implementation context and readiness

In February and March of 2015, our research team made in-person, 2-day baseline visits to each of the three health systems. On the first day, we conducted observations of patient care areas, collected artifacts, and received an orientation to the clinical workflow. We also conducted and audio-recorded semi-structured interviews of 2 each of local primary care clinicians, implementation team members, and health system leaders in each system. At the end of the first day, we debriefed the experience amongst ourselves and then with the system’s dedicated physician champion. We summarized our thoughts in field notes and used the insight to structure an implementation team workshop on the second day. This workshop included an overview of SDM, practice with the SCCA, and a facilitated discussion and plan for implementation, guided by the Normalization Process Toolkit [44]. After completion of the baseline visit, we administered the patient, clinician, implementation team, and leadership surveys, electronically first and then in paper format to non-responders by on-site study coordinators.

##### Implementation work and outcomes

After collection of the baseline data, we enrolled implementation teams from all three systems into a web-based project management site (Basecamp) to facilitate communication and learning. We scheduled occasional teleconferences with and between the three systems to discuss key challenges and brainstorm solutions. At approximately 6 months of follow-up (Aug-Sep 2015), we returned to each health system. At these visits, we conducted follow-up interviews, conducted and recorded a 90-min focus group with the implementation team, and video-recorded 2 simulated clinical encounters by which clinicians used the tool as it existed in their current workflow and practice. We also repeated the patient, clinician, implementation team, and leadership surveys at this time and again at 18 months of follow-up using the same procedures as at baseline. Throughout the study period we collected monthly Google Analytic reports of Statin Choice usage by health system IP address overall and normalized according to the number of primary care clinicians. We shared these comparative reports with the implementation teams through the Basecamp site on a monthly basis. We kept abreast of the activities and emergent implementation strategies that the on-site implementation teams were pursuing and used this information to interpret any changes in measured usage.

#### Innovate, develop, and engage

Based on key findings from the Assess phase, we worked in partnership with the 3 systems to modify the tool for more seamless clinical integration (e.g. by working with the developer of the tool to enable auto-population of clinical parameters required by SCCA and EMR integration) and to develop procedures and materials that could reduce the work of implementation. We took note of and reached out to stakeholder groups and networks that could devolve the strategy early in this process.

### Analysis

#### Implementation context and readiness

We conducted separate Assess phase analyses for each of the two stages. In the first stage, we used baseline data from surveys, interviews, and on-site observations to characterize the system contexts and to estimate the feasibility, appropriateness, and acceptability of implementation in each setting.

For the qualitative analysis, a team of two analysts (AL and KB) coded all qualitative data independently and in duplicate according to pre-defined codes of the Consolidated Framework for Implementation Research (CFIR) [33] within the domains of Outer Setting, Inner Setting, and Individual Characteristics, and by health system. We searched for CFIR codes that seemed to distinguish the health systems, discussed the emerging data as a research team at regular meetings, and resolved any discrepancies.

Quantitative outcomes at baseline were calculated with standard descriptive statistics and included patient involvement in decisions, clinician exposure to and beliefs about SDM and the SCCA and its baseline usage, implementation team members’ confidence in implementation success, and system leadership’s perception of its relative priority.

Over a series of research team meetings and discussions, we merged insights from the two forms of data within pre-defined outcome domains of implementation feasibility, appropriateness, and acceptability and formulated “contextual summaries” of the three health systems.

#### Implementation work and outcomes

In the second stage of the Assess phase, we analyzed the work of implementation in each system. Here, a team of two analysts (KB, SD), after establishing agreement on a pilot transcript and with the lead investigator, used pre-defined codes (e.g. coherence, cognitive participation, collective action, and reflective monitoring work) from Normalization Process Theory [38] to analyze all the 6-month qualitative data, taking note of how the various types of work were allocated to affect the feasibility, acceptability, and appropriateness of implementation. They worked independently and in duplicate, resolving discrepancies by consensus and with support from the lead investigator. For the quantitative analysis, we evaluated each system independently, looking for changes in survey outcomes over time within each system and not prioritizing cross-system comparisons. We used a Cochran Armitage trend test for categorical variables and compared continuous outcomes with a Kruskal-Wallis test. We triangulated all this insight with follow-up results from the Google Analytic usage reports and in consideration of the individual systems’ baseline contexts.

## Results

### Assess

#### Implementation context and readiness assessment

Qualitatively, we found few clear distinctions between the Outer Settings or Individual Characteristics of the three systems (as described in the CFIR [33]), although their Inner Settings differed in regards to organizational size, structure, maturity, culture, and capacity for collaboration, communication, and implementation. Survey measures were mostly consistent with the qualitative insight, suggesting favorable climates overall, but with modest differences between systems. Summaries of the qualitative and quantitative data used to develop the contextual summaries—including patient and clinician demographics—are presented in Additional file 2. Here we present only the global, integrated assessments of implementation feasibility, acceptability, and appropriateness in Table 3.

**Table 3:** Integrated context and readiness assessments

Global, Integrated Implementation Context and Readiness Assessments			
	System 1	System 2	System 3
Representative Stakeholder Quote	“organic we’re good, process…not so good”	“educate, that’s what we do”	“we’re turning into something bigger”
Relative Feasibility	+	+++++	+++
Relative Acceptability	+++	++	+++++
Relative Appropriateness	++	++	++++
*“+” Represents global judgements within a possible range of “+” to “+++++”*			

#### Implementation work and outcomes assessment

**Fig. 3:**
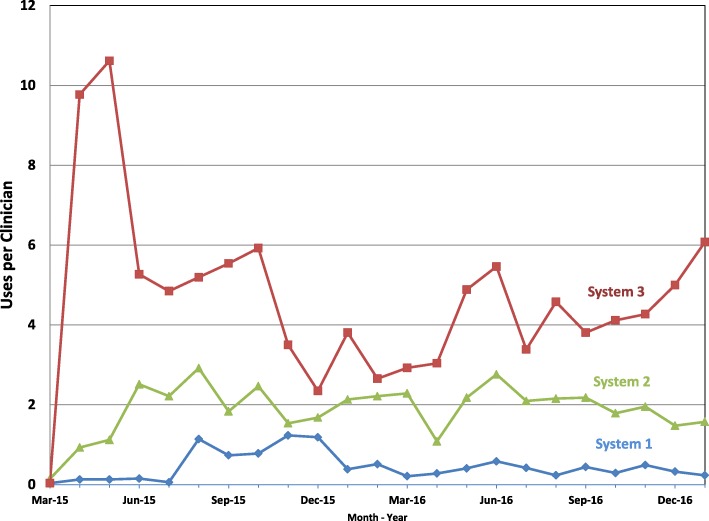
Average monthly per clinician SCCA usage over time across the 3 health systems

Across all 3 systems, self-reported and measured usage of the SCCA increased throughout the study period. This usage increased most dramatically and sustainably in system 3 and to a lesser extent in system 2. Usage in system 1 increased modestly for a period but was not sustained. (Fig. 3) Video-recorded encounter simulations suggested that clinicians in all systems used the tool from within their own workflow with high fidelity and in a way known to promote SDM. We found no significant changes in survey measures of appropriateness (e.g. SDM beliefs) or tool acceptability (e.g. SCCA beliefs), although there were trends suggesting acceptability of the tool and usage increased over time in a correlated fashion. For a full summary of these and other implementation outcome data organized by system, see Additional file 2. Here we focus on describing the work that was needed to build potential for implementation and how it was perceived to influence these outcomes.

Specifically, we found that effort was allocated to optimize the feasibility, acceptability, and appropriateness of implementation in complex and interdependent ways. Potential was achieved most readily, it seemed, when resources were directed toward improving the implementation outcome domains judged weakest in the baseline assessments. Often, different types of work were undertaken for different but synergistic purposes and by or for the benefit of different but equally critical stakeholders.

For example, coherence work was often needed to improve perceptions of the appropriateness of implementation among both organizational and IT leadership and clinicians. For leaders, this often amounted to understanding the “pay-off” that could be expected from the investment of resources. Importantly, this was not something our team was able to provide or predict at project outset, but which leadership felt would have only increased its desire to engage:

“I guess I was expecting this to be kind of more time and mental energy consuming than it was. If I only knew how straight forward that rule build [in the EMR], that was the majority of the build, if I knew how straight forward that was I would have been less kind of feeling like it was a lot coming towards me in the beginning.” (IT Leader, System 1)

Clinicians, on the other hand, came to make sense of the tool’s appropriateness by judging whether it had been endorsed by organizational leadership and by assessing its impact on clinical workflow and patient care. Systems influenced this perception by having respected physician leaders communicate the SCCA’s status as an organizational priority and by explicitly demonstrating the “reason for SDM,” despite sometimes conflicting practice guidelines.

“I knew there was support from the CEO that this was a good thing to do; I guess that was what really validated it, when we did the brochure that went out to the [clinics], the one that went out to the community—that really reinforced that this wasn’t just any old thing, it was something we valued as an organization.” (Clinician, System 3)

The feasibility of implementation was largely determined by the capability of the multi-disciplinary implementation team to communicate these and other messages across the organization. This was because success was predicated on the collaboration of multiple departments and the enrollment of support across many clinics. Cognitive participation work was especially salient in that regard and systems that were small (System 3) or had long-standing procedures for working together and communicating efficiently (System 2) were at an advantage, provided they accessed this potential. In the case of System 2, that amounted to using existing provider meetings and educational infrastructures and standing teams. In the case of System 3, it amounted to use of an existing meeting and the addition of one implementation team meeting to allocate responsibility.

“We also…tried to project it to other clinicians in our [system] and did it by different means, including the quarterly meetings and then the continued education meetings, also in our clinic meetings, and some meetings in between too.” (Leadership, System 2)

Where feasibility was low, organizations had to devote resources to increasing communication and teamwork potential if they wanted to succeed. Notably, System 1, which was judged lowest for feasibility at outset, allocated little effort to improving capability in this domain. This was partly because implementation was judged to be less appropriate here. Indeed, the organization had just finished implementing another intervention aimed at a similar goal.

“Like I said, I’ve had very little involvement. We haven’t been asked to do anything…maybe those conversations have occurred and I’m just not aware of them.” (Implementation Team Member, System 1)

By not directing effort to improve feasibility or appropriateness, the value of efforts to improve acceptability might have been reduced. For example, significant collective action work was done by information technology within System 1 to fully integrate the SCDA into the electronic medical record and thus increase acceptability by clinicians. Unfortunately, at the post-implementation visit, no one on the implementation team was aware this work had been done and no one had trained the clinicians on how to use the tool. This limited uptake of the tool in System 1—with only 18% of clinicians reporting routine usage at 6 months compared to 38 and 69% in Systems 2 and 3, respectively—and, as shown in Fig. 3, usage was not sustained.

As it turned out—and consistent with prior studies of the SCCA—only a small amount of work was needed to adequately train the clinicians on how to use the tool, but this work was essential and it had to be delivered reliably. When the tool was demonstrated for a few minutes (either in person or via email-delivered video) by an internal expert as it existed in the clinicians’ own electronic workflow, it seemed to help clinicians overcome the coherence work of understanding what the tool did and how it could be used efficiently. Once the clinicians tried the tool with one or a few actual patients, they often found it changed their practice, which seemed to increase acceptability. This was consistent with survey data showing correlation trends between usage and positive SCCA beliefs (**see** Additional file 2).

“It [was, at first] something that I used out of—just out of respect and appreciation for colleagues that say, we wanna try this out. It had nothing to do with my desire to change the way I implemented statin medications. I felt like we were doing fine. I think that’s what most providers will say is, I’m doing fine. What it has shown is just a greater understanding of patient awareness overall. We know they made a good educated choice….The best advice I could give [to another provider] is ‘be open –minded and try it more than once before you decide whether or not it’s something you’re gonna do.” (Clinician, System 3)

Real world usage of the SCCA was a key factor in helping clinicians to identify a relative advantage for the tool, and it was the most important example of reflexive monitoring work we found. When early adopters were put in a position of using the tool, they could further enhance acceptability and facilitate spread through informal peer to peer communication.

“I think the best thing, though, is whenever it’s mentioned at these quarterly meetings, because then it puts it in the forefront…because one of their partners may say, ‘You mean you’ve been using this tool?,’ ‘Sure,’ and they can show ‘em…then you’ve got two people, then you’ve got four, and it just migrates.” (Leadership, System 2)

In short, and perhaps because the tool was developed through a user-centered design approach [3] and was known to be well-accepted by clinicians, we found most of the work required to implement the SCCA was dissemination-related work done by the organization. When key efforts were not taken to address parts of this work, success was lessened.

“I don’t think that piece of it—just putting the [SCCA] in the right spot and bringing the data from our [EMR] into that, I’m guessing was a week, week-and-a-half, not horrible. The deployment, actually getting it out to the other docs, is probably much bigger, the training and tracking issues of hey, did you know there’s this new tool, and using that. Now, having said that, that’s often very critical to get adoption, otherwise it just sits out there and languishes.” (Leadership, System 2)

For a complete summary of the strategies systems used to reduce the work of implementation and prevent the SCCA from “languishing” see Table 4. There we organize strategies by their judged value and according to the type of work they were perceived to reduce and the stakeholders and outcomes they were judged to influence. Note that all judgements are global impressions of the authors that have been confirmed with staff at each participating system.

**Table 4:** Strategies taken to increase potential for implementation of the SCCA

Strategy Taken	Work Type (NPT) and Target Stakeholder	Outcome	Systems Pursuing (1, 2, 3)	Perceived Value
Introductory, Facilitated Implementation Team Workshop	Coherence of Implementation Team	Feasibility, Acceptability, Appropriateness	1, 2, 3	+++++
Basecamp Learning Community and Resource Access	Collective Action of Implementation Team	Feasibility, Appropriateness	1, 2, 3	+
Facilitated Cross-organization Conference Calls	Collective Action of Implementation Team	Appropriateness	1, 2, 3	+
Dedicated or de facto project manager	Collective Action of Implementation Team	Feasibility	2, 3	+++
Implementation team meetings	Collective Action of Implementation Team	Feasibility	2, 3	+++
Demonstration of tool at clinician meetings/conferences by local champion	Coherence of Clinicians	Acceptability	2, 3	+++++
Video tutorial email to providers of tool in local EMR	Coherence of Clinicians	Acceptability	2	+++++
Personal letter to clinicians from leadership in support of SDM and tool	Cognitive Participation of Clinicians	Appropriateness	1	+
External marketing in newsletter of organization	Coherence of Clinicians	Appropriateness	3	+++
Engagement of EMR vendors to support local integration	Coherence and Collective Action for Information Technology	Feasibility and Acceptability	1, 2, 3	+++++
Integration of tool to EMR to auto-populate patient characteristics	Collective Action for Clinicians	Feasibility and Acceptability	1, 2, 3	+++++
*“+” Represents global judgements within a possible range of “+” to “+++++”*				

### Innovate, develop, and engage

Throughout the Assess phase, we took note of opportunities for modifying the SCCA to improve its fit in diverse contexts. Most notably, we worked with system IT experts to develop strategies that could integrate the tool into the electronic workflows of each system. We coordinated necessary changes with the tool’s developer to permit this innovation. Through interaction with system stakeholders, we were connected to their EMR vendors and learned of further ways to optimize the tool for widespread use (e.g. by developing clinical program summaries that could be posted on EMR vendors’ customer web spaces). After synthesizing insights from the Assess phase, we considered the value of strategies that were taken and we engaged system stakeholders in considering other strategies that would have been helpful. We collected these strategies into a toolkit (Table 5 and online at **shareddecisions.mayoclinic.org/resources/statin-choice-toolkit****/**) that could guide stakeholders in other settings in assessing their own contexts, determining the appropriateness of implementation, and strategically targeting efforts to accomplishing the work of implementation. We also put a link to this toolkit on the web-based SCCA itself so that “lone users” would be equipped to champion implementation in their own organizations.

**Table 5:** Strategies identified for inclusion in the SCCA implementation toolkit

The Statin Choice Conversation Aid Implementation Toolkit		
Strategies of Judged Value	Work Type and Target Stakeholder	Outcome
Targeted Context Self-assessments	Coherence of Leadership, Implementation Team, Information Technology	Understanding of Appropriateness
Leadership Resource Estimates and Testimonials	Coherence of Leadership	Appropriateness
Pre-Recorded Preparatory Webinar for Implementation Team	Coherence of Implementation Team	Feasibility, Acceptability, and Appropriateness
Implementation Team Manual and Meeting Agendas	Collective Action of Implementation Team	Feasibility
Internal Marketing Material Examples	Coherence of Clinicians	Appropriateness
EMR Vendor-specific Integration and Coding Guides	Coherence and Collective Action of Information Technology	Feasibility and Acceptability
SDM Presentation Templates	Coherence of Clinicians	Acceptability and Appropriateness
SCCA Video Tutorial Example	Coherence of Clinicians	Acceptability

Key components of the toolkit include an Implementation Team Manual that guides organizations through a step-by-step process of forming their team, learning together, conducting assessments, and organizing activities, as well as accompanying webinars and video demos that the team can watch together and replicate and/or disseminate as appropriate. Toolkit materials were designed to be highly practical to address the many practical barriers we identified. This included email language that could be used to communicate the value proposition to system leadership and EMR integration support documents that described the very technical language and codes needed to integrate the tool electronically.

## Discussion

### Discussion

We conducted a theory-guided, formative evaluation of the work of implementing a conversation aid designed to promote SDM in 3 different health systems and used insights from this evaluation to develop an implementation toolkit. The conceptual orientation of our study contributes to the field of implementation science by strategically unifying the work of others. Specifically, it adds to Bradley et al.’s model for scale-up [30] consideration of the work stakeholders must do achieve implementation [38] and the outcomes [36] their efforts must optimize for adoption and uptake to occur. It also complements the work of others [45] by serving as an example of how formative evaluations in index settings can be coherently oriented [25, 46, 47] to develop bundled implementation strategies and toolkits that can be broadly disseminated.

Although we focused our assessment on the implementation outcome domains of feasibility, acceptability, and appropriateness, we do not intend to imply that other outcomes described by Proctor et al. [36] are not relevant (e.g. adoption, maintenance, fidelity, and sustainability). Rather, we operated under the assumption that optimization of these “early phase” outcomes would be most important for enabling the unsolicited uptake that is required for scale-up and spread [48]. It is also not our impression that the toolkit we created and the strategies it comprises will necessarily be appropriate for facilitating the scale of all patient decision aids, conversation aids, or other interventions to directly or indirectly promote SDM. Rather, we believe that any SDM intervention is likely to have a unique set of barriers and facilitators to implementation. These should be discovered in an analogous process. The SCCA was an intervention developed through a user-centered design approach and we were already familiar with its effectiveness and its potential for sustained use with fidelity. This might have made the tool uncommonly poised for a targeted evaluation of the barriers to scale-up and spread. Still, we identified situations in which systems were not ideally positioned to implement the tool. Our resulting context self-assessments may provide value in helping to distinguish systems that are ready for this work. These assessments are, conceptually, measuring constructs of readiness for change [49], although they guide stakeholders in considering the real-world, intervention-specific factors (such as information technology bandwidth and the availability of standing clinician meetings) that comprise the implementation climate.

Along these lines, it is also worth noting that the Agency for Healthcare Research and Quality (AHRQ) developed a toolkit to support SDM implementation broadly in 2015 [50]. To our knowledge, no theory was employed in organizing its structure or contents. Its effectiveness in overcoming the many known barriers to SDM implementation [35, 37, 51, 52] has also not been evaluated and its applicability to any single SDM intervention (such as the SCCA) is likely limited. Indeed, our present research and experience suggests that more targeted toolkits, designed to counteract the unique and intervention-specific barriers to implementation (and nothing more) will be more effective in facilitating scale-up of any single SDM intervention. These targeted toolkits could (and perhaps should), however, be paired with interventions like the AHRQ toolkit that are intended to promote SDM broadly.

### Strengths and limitations

The scope of our implementation context and process evaluation is a major strength of this study. Indeed, we used multiple methods to rigorously assess multiple levels and perspectives across the entire breadth of 3 different healthcare systems in different regions of the United States. The consistency of insights across these settings increases our confidence that the results are representative and that our conceptual rationale has value. The study also has several limitations. Because the quantitative measures were not validated and did not prove very sensitive to change, most judgements were based on qualitative data. Our study was not designed to definitively establish mechanistic relationships between the constructs we considered. Rather it should be viewed as exploratory and as a heuristic for orienting future thinking and research related to implementation strategy development and the scale-up of SDM interventions.

### Practice implications and future directions

Related to this, our biochemistry-informed “work reduction model” (Fig. 2) may be useful to practitioners and researchers who are considering developing toolkits for dissemination. Indeed, if the expectation is that toolkits will be passively picked up to facilitate spread and affect change, our model proposes these interventions should be constructed empirically and designed to reduce the early-phase work stakeholders must do to achieve implementation. Before such strategies can be designed reliably, however, it is necessary to further explore the relationships between the constructs we propose (e.g. the work of implementation and the outcomes it achieves). We found that task challenging. To that end, the development and testing of better measures and the use of methods that can define mechanistic pathways would be helpful. Then, for example, future research could compare the effectiveness of scale-up strategies based on theories of work reduction against those that are not.

## Conclusion

We developed a multi-component toolkit for facilitating the scale and spread of an intervention to promote SDM across clinical settings. The theory-based approach we employed in designing the toolkit may have value in orienting the development of multi-component toolkits and other implementation strategies aimed at facilitating the efficient scale of interventions.

## Additional files

Additional file 1: Study Measures and Guides. (PDF 2683 kb)

Additional file 2: Quantitative and Qualitative Data Summaries. (PDF 1866 kb)

## Abbreviations

ACC/AHA: American College of Cardiology/American Heart Association
AHRQ: Agency for Healthcare Research and Quality
AIDED: Assess, Innovate, Develop, Engage, Devolve
BTS: Breakthrough Series
CEO: Chief Executive Officer
CFIR: Consolidated Framework for Implementation Research
EMR: Electronic medical record
IHI: Institute for Healthcare Improvement
IT: Information Technology
NPT: Normalization Process Theory
SCCA: Statin Choice Conversation Aid/Statin Choice Decision Aid
SDM: Shared decision making
