# Supplementary material for: Developing a toolkit to implement the Statin Choice Conversation Aid at scale: application of a work reduction model
Source: BMC Health Serv Res. 2019 Apr 24;19:249. doi: 10.1186/s12913-019-4055-8 (PMC6480421; doi:10.1186/s12913-019-4055-8)
Supplement: Supplementary file 1 — Study Measures and Guides. (PDF 2683 kb) [file 12913_2019_4055_MOESM1_ESM.pdf]

Health System Logo

Patient Collaboration Survey

Your health system is participating in a research study of the Mayo Clinic Care Network. The purpose of this research study is to better understand and improve the way patients and clinicians work together to make health care decisions.

This is a research study and your participation is completely voluntary. To respect your confidentiality, this survey does not collect any identifying information. No one will be able to connect you to your responses in any way and your responses will not affect the care you receive from your health system. By completing this brief survey you agree to participate in this research study.

Your answers to these questions will help direct efforts to improve the quality of care for patients in your health system and generate research knowledge that can improve care for many others.

If you have any questions or comments about this survey or study, please call: 1-507-XXX-XXXX

Thank you for answering these questions!

© Mayo Clinic Care Network Learning and Research Collaborative

Some questions about your visit:

For each of these questions, please circle one.
Thinking about the visit you had with your health care provider today…

1. **How much effort was made to help you understand your health issues?**

No effort at all	0	1	2	3	4	5	6	7	8	9	Every effort was made

2. **How much effort was made to listen to the things that matter most to you about your health issues?**

No effort at all	0	1	2	3	4	5	6	7	8	9	Every effort was made

3. **How much effort was made to include what matters most to you in choosing what to do next?**

No effort at all	0	1	2	3	4	5	6	7	8	9	Every effort was made

Some questions about you:

1. **What is your gender? [please check ☑ one]**

☐$_0$ Female
☐$_1$ Male

2. **What is your age?**

_______ years

3. **Are you of Hispanic or Latino origin or descent? [please check ☑ one]**

☐$_0$ Yes, Hispanic or Latino
☐$_1$ No, not Hispanic or Latino

4. **What is your race? [please check ☑ one or more]**

☐$_1$ White
☐$_2$ Black or African American
☐$_3$ Asian
☐$_4$ Native Hawaiian or Other Pacific Islander
☐$_5$ American Indian or Alaska Native
☐$_6$ Other

Thank you for answering these questions! Please return this survey to the front desk before leaving!

System Number:__ __ __ Practice Number:__ __ __

Health system logo

Primary Care Clinician Survey

The Statin Choice Implementation Project

These questions seek to understand how you make decisions with your patients about starting a statin for primary cardiovascular prevention.

As this is a research study, your participation is voluntary. Completing this survey indicates your agreement to participate in this research study. To respect your privacy, we will remove any information that may allow us to connect you to your responses upon receiving your completed questionnaire.

Your answers to these questions may help direct efforts to improve the quality of care for patients in your health system and generate research knowledge that may improve care for many others.

If you have any questions or comments about this survey or study, please call: 1-507-XXX-XXXX

Thank you for answering these questions!

© Mayo Clinic Care Network Learning and Research Collaborative

Some Questions About You:

1. What is your degree within your healthcare profession? [please check ☑ one]

☐1 MD/DO
☐2 PA
☐3 NP
☐4 RN
☐5 Other (specify) ____

2. What is your gender?

☐0 Female
☐1 Male

3. What is your age?

______ years

4. Are you of Hispanic or Latino origin or descent?

☐0 Yes, Hispanic or Latino
☐1 No, not Hispanic or Latino

5. What is your race? [please check ☑ one or more]

☐1 White
☐2 Black or African American
☐3 Asian
☐4 Native Hawaiian or Other Pacific Islander
☐5 American Indian or Alaska Native
☐6 Other

Some Questions About Shared Decision Making:

When patients and clinicians work together to share information about the available options and arrive at a decision, they are said to be practicing shared decision making. Please state the extent to which you disagree or agree with the following statements. [please check ☑ one response for each statement]

6. In general, I believe that shared decision making…	Strongly Disagree ↓	Disagree ↓	Slightly Disagree ↓	Slightly Agree ↓	Agree ↓	Strongly Agree ↓
a. …improves patient satisfaction	☐	☐	☐	☐	☐	☐
b. …increases health care costs	☐	☐	☐	☐	☐	☐
c. …improves patient outcomes	☐	☐	☐	☐	☐	☐
d. …is promoted by my health system/organization	☐	☐	☐	☐	☐	☐
e. …makes practice less evidence-based	☐	☐	☐	☐	☐	☐
f. …is too rigid to be applied to most patients	☐	☐	☐	☐	☐	☐
g. …is the typical way I make decisions with patients	☐	☐	☐	☐	☐	☐
h. …is the typical way most clinicians make decisions with patients	☐	☐	☐	☐	☐	☐

System Number: __ __ __ Practice Number: __ __ __ Clinician Number: __ __ __

Date: __ /__/ __ Cycle: 0

Some Questions About the Statin Choice Decision Aid:

The Statin Choice Decision Aid helps appropriate patients and clinicians make a decision together about using statins to prevent cardiovascular events. It presents tailored risk estimates, potential harms, costs, and inconveniences.

Note that, for this survey, we consider "appropriate patients" for the Statin Choice Decision Aid to be adults that have never had a cardiovascular event, are not diabetic, and have LDL cholesterol <190 mg/dL.

Please answer the following questions about the Statin Choice Decision Aid.

7. What is your level of exposure to the Statin Choice Decision Aid? [please check ☑ one]

☐1 I have never heard of it ➔ Thank you for your time! **You may stop now**. Please return this survey to your clinic manager.

☐2 I have heard of it but have not seen any patients that would be considered appropriate
☐3 I have heard of it but have not used it with any patients that would be considered appropriate
☐4 I have used it once or occasionally with patients that would be considered appropriate
☐5 I use it routinely with patients that would be considered appropriate

8. The clinical guidelines for use of statins for cardiovascular risk reduction recently changed. They now encourage therapy decisions be based on 10 year cardiovascular risk calculations and that statins be prioritized among all lipid lowering agents. What role does this play in your use of the Statin Choice Decision Aid? [please check ☑ one]

☐1 The guidelines have made me more likely to use the Statin Choice Decision Aid
☐2 The guidelines have had no affect on whether I use the Statin Choice Decision Aid
☐3 The guidelines have made me less likely to use the Statin Choice Decision Aid

System Number: __ __ __ Practice Number: __ __ __ Clinician Number: __ __ __

Date: __ /__/ __ Cycle: 0

Please state the extent to which you disagree or agree with the following statements <u>about the Statin Choice Decision Aid</u>. [please ☑ one response for each statement].

9. In general, I believe the Statin Choice Decision Aid…	Strongly Disagree	Disagree	Slightly Disagree	Slightly Agree	Agree	Strongly Agree
a. …improves my ability to have a shared decision making conversation with my patients	☐	☐	☐	☐	☐	☐
b. …is too complex or cumbersome for practical use	☐	☐	☐	☐	☐	☐
c. …improves the quality of care delivery in my practice	☐	☐	☐	☐	☐	☐
d. …is used by most clinicians like me in this health system	☐	☐	☐	☐	☐	☐
e. …is readily available for me to use if and when I choose	☐	☐	☐	☐	☐	☐

10. We are looking for ways to improve the Statin Choice Decision Aid. If you have one suggestion for how we could improve the <u>Statin Choice Decision Aid</u>, please state it here:
____

____

11. We want to make it easy for you to use the Statin Choice Decision Aid. If you have one suggestion for how we could change your <u>practice environment or context</u> to make it easier for you to use the Statin Choice Decision Aid, please state it here:
____

____

Thank you for completing this survey!

System Number: __ __ __ Practice Number: __ __ __ Clinician Number: __ __ __

Date: __ /__/ __ Cycle: 0

Clinician Semi-structured Interview Guide: SCIP Phase 1

INTRODUCTION:

Thank you for taking the time to talk with me today. Our research team is working with your health system to implement the Statin Choice Decision Aid into primary care practice and to study the factors that impact this process. The Statin Choice Decision Aid is a tool that patients and clinicians use together to have a shared decision making conversation about whether to start a statin for primary prevention of cardiovascular events. As a primary care clinician, you have special insight into factors that can affect the up-take and use of the Statin Choice Decision Aid and we are very interested in understanding your perspective.

In this interview, I will be asking you questions about your thoughts and experiences. I want to assure you that your participation is voluntary and that you can choose to stop at any time. If a question makes you uncomfortable or you do not want to answer, that is fine; you do not have to. To respect your privacy, all of your responses will be held in confidence and no one will be able to connect you to any of the things you say.

You should feel open to express your thoughts and experiences honestly and to ask for clarification if any questions seem confusing.

Okay, do you have any questions before we get started?

WARM-UP CHAT (unstructured):

Okay, great. So, first I'd just like to learn a little bit about you. Please tell me about your scope of practice in this health system. How long have you been here? Have you participated in any research studies before? Etc…

Transition: Okay. Thank you. This is all very helpful to me.

SDM CULTURE:

Every organization has a unique culture and way of doing things. I would like to hear your perspective on the culture of this health system. How would you describe the culture of this health system?

Probes:

- What are its major priorities?
- How does this health system work to make care more patient-centered?
- Have you ever heard of the concept of shared decision making?
- How does this health system work to encourage shared decision making?

Transition: I understand. This is very helpful.

CAPACITY FOR CHANGE:

As a primary care clinician, I know you're aware of the challenges involved in changing behaviors and routines. These challenges can be even greater when trying to promote change at an organizational level. I am interested in hearing your perspective on this health system's potential for change. How would you describe the capacity of this health system to make system-wide changes in practice?

Probes:

- Please describe an example of a time when this health system tried to make a change and succeeded
- Please describe an example of a time when this health system tried to make a change and failed
- Please describe your perception of the factors that are important in making change happen in this health system in general

Transition: I understand. This is excellent information and very helpful to us.

MAJOR FACILITATORS TO SCDA:

For the rest of the interview, I'd like to focus specifically on the Statin Choice Decision Aid. The Statin Choice Decision Aid is a web-based tool that patients and clinicians can use together to facilitate a shared decision making conversation about starting a statin for primary cardiovascular risk prevention. If you have seen or used this tool, I would like you to reflect on your experiences when answering these questions. If you have not had exposure to the tool, I would still like to hear your thoughts about what you imagine using it would be like. How familiar are you with the Statin Choice Decision Aid? [note response] In your opinion, what are the factors that motivate clinicians to use this tool?

Probes:

- Please describe a feature of the tool that makes it valuable to you
- Please describe a feature of your context or health system that helps to promote usage of the tool

Transition: Okay. This is good for us to know.

MAJOR BARRIERS TO SCDA:

Now I'd like to ask the same questions but focus on the things that impede usage of the Statin Choice Decision Aid. In your opinion, what are the factors that cause clinicians not to use this tool?

Probes

Probes:

- Please describe a feature of the tool that makes it difficult to use or reduces its value to you
- Please describe a feature of your context or health system that inhibits usage of the tool

I understand. This has all been very helpful. I think that's all I need unless you have any other comments or questions.

Okay, thank you again for taking the time to chat with me about this.

Rater :   System ID:

Date :   Clinician ID:

SCIP: Statin Choice Fidelity Scale

**Baseline Risk**				
**1.) Did the clinician describe the risk as a natural frequency** *(e.g. "of 100 people like you…")*?	0☐	N	1☐	Y
**2.) Did the clinician describe the time horizon for risk of having a heart attack?** *(e.g. "…in the next ten years")*	0☐	N	1☐	Y
**3.) Did the clinician describe the risk graphically?**	0☐	N	1☐	Y
**Risk Reduction (from Statins)**				
**4.) Did the clinician describe risk reduction as a natural frequency** *(e.g. "if 100 people, like you, take the medication, 10 will still have a hear attack, and 5 people will avoid a heart attack")*?	0☐	N	1☐	Y
**5.) Did the clinician describe the time horizon for the risk reduction?** *(e.g. ". . . within the next 10 years")*	0☐	N	1☐	Y
**6.) Did the clinician describe the risk reduction graphically?**	0☐	N	1☐	Y
**Downsides**				
**7.) Did the clinician describe the duration of taking the statin?** (e.g. for a long time or maybe forever)	0☐	N	1☐	Y
**8.) Did the clinician describe at least one of the common side effects** *(e.g. nausea, diarrhea, constipation, muscle aching/stiffness, liver blood test goes up, muscle and kidney damage)*	0☐	N	1☐	Y
**9.) Did the clinician describe the frequency of at least one of the side effects** *(e.g. 5 in 100, 2 in 100, 1 in 20,000)*	0☐	N	1☐	Y
**Cost**				
**10.) Did the clinician describe the cost of the medication** *(e.g. with or without insurance)*?	0☐	N	1☐	Y
**TOTAL POINTS**	Total "Yes" : --			
OTHER				
**11.) Did the clinician discuss diabetes care, exercise, or lifestyle changes?**	0 ☐	N	1 ☐	Y
**12.) What decision was made today?**	0 ☐	Take or continue statin.	1 ☐	Not take or stop statin
	2 ☐	Prefer to decide at some other time		
**13.) Comments:**				

Implementation Team Survey

The Statin Choice Implementation Project

Your health system is participating in a research study of the Mayo Clinic Care Network. The purpose of this questionnaire is to obtain information from you about your experiences in trying to implement the Statin Choice Decision Aid into routine primary care practice.

This is a research study and your participation is completely voluntary. To respect your confidentiality, this survey will be de-identified as soon as you return it. No one will be able to connect you to your responses in any way. By completing this survey you agree to participate in this research study.

Your answers to these questions will help direct efforts to improve the quality of care for patients in your health system and generate research knowledge that can improve care for many others.

If you have any questions or comments about this survey or study, please call: 1-507-XXX-XXXX

Thank you for answering these questions!

© Mayo Clinic Care Network Learning and Research Collaborative

We want to understand your thoughts and experiences. Please indicate the extent to which you agree or disagree with the following statements. [please check ☑ one]

Some Questions About Your Health System:

1. Participating in healthcare research is a priority of this healthcare system.

   ☐1 Strongly disagree
   ☐2 Moderately disagree
   ☐3 Slightly disagree
   ☐4 Slightly agree
   ☐5 Moderately agree
   ☐6 Strongly agree

2. Engaging patients in shared decision making is a priority of this health system.

   ☐1 Strongly disagree
   ☐2 Moderately disagree
   ☐3 Slightly disagree
   ☐4 Slightly agree
   ☐5 Moderately agree
   ☐6 Strongly agree

3. Implementing the Statin Choice Decision Aid is a priority of this health system.

   ☐1 Strongly disagree
   ☐2 Moderately disagree
   ☐3 Slightly disagree
   ☐4 Slightly agree
   ☐5 Moderately agree
   ☐6 Strongly agree

4. When this health system decides that change is needed, it is effective at making it happen.

   ☐1 Strongly disagree
   ☐2 Moderately disagree
   ☐3 Slightly disagree
   ☐4 Slightly agree
   ☐5 Moderately agree
   ☐6 Strongly agree

Some Questions About Implementing the Statin Choice Decision Aid:

5. I believe our team has the necessary resources and support from this health system to succeed in implementing the Statin Choice Decision Aid.

   ☐1 Strongly disagree
   ☐2 Moderately disagree
   ☐3 Slightly disagree
   ☐4 Slightly agree
   ☐5 Moderately agree
   ☐6 Strongly agree

6. I believe our team has the necessary resources and support from the Mayo Clinic research team to succeed in implementing the Statin Choice Decision Aid.

   ☐1 Strongly disagree
   ☐2 Moderately disagree
   ☐3 Slightly disagree
   ☐4 Slightly agree
   ☐5 Moderately agree
   ☐6 Strongly agree

System Number: __ __ __ Team Number: __ __ __ Member Number: __ __ __

Date: __ /__/ __ Cycle: 0

7. Collaborations with other health systems in the Mayo Clinic Care Network will help our team succeed in implementing the Statin Choice Decision Aid.

☐1 Strongly disagree
☐2 Moderately disagree
☐3 Slightly disagree
☐4 Slightly agree
☐5 Moderately agree
☐6 Strongly agree

8. I am confident that our implementation strategy will succeed in implementing the Statin Choice Decision Aid.

☐1 Strongly disagree
☐2 Moderately disagree
☐3 Slightly disagree
☐4 Slightly agree
☐5 Moderately agree
☐6 Strongly agree

9. I believe that now is the right time to implement the Statin Choice Decision Aid.

☐1 Strongly disagree
☐2 Moderately disagree
☐3 Slightly disagree
☐4 Slightly agree
☐5 Moderately agree
☐6 Strongly agree

We want to know a little bit about your background. For the following questions, please ☑ one.

<u>Some Questions About You:</u>

10. Which best describes your professional background?

☐1 Clinical practice (MD, DO, NP)
☐2 Nursing care (RN, LPN)
☐3 Allied health professions (pharmacy, respiratory therapy, physical therapy)
☐4 Information technology
☐5 Administration and/or management
☐6 Social work and/or case management
☐7 Quality improvement and/or system engineering
☐8 Other (specify)
____
____

11. What is your gender?

☐0 Female
☐1 Male

12. Are you the designated MCCN Learning and Research Collaborative Liaison for your health system?

☐0 No
☐1 Yes
☐2 Unsure

Shared decision making is an approach to clinical decision making that patients and clinicians engage in together. We would like to know some of your thoughts about shared decision making. Please state the extent to which you disagree or agree with the following statements. [please check ☑ one response for each statement]

13. In general, I believe that shared decision making…	Strongly Disagree	Disagree	Slightly Disagree	Slightly Agree	Agree	Strongly Agree
a. …improves patient satisfaction	☐	☐	☐	☐	☐	☐
b. …increases health care costs	☐	☐	☐	☐	☐	☐
c. …improves patient outcomes	☐	☐	☐	☐	☐	☐
d. …is promoted by my health system/organization	☐	☐	☐	☐	☐	☐
e. …makes practice less evidence-based	☐	☐	☐	☐	☐	☐
f. …is too rigid to be applied to most patients	☐	☐	☐	☐	☐	☐
g. …is the typical way I make decisions with patients	☐	☐	☐	☐	☐	☐
h. …is the typical way most clinicians make decisions with patients	☐	☐	☐	☐	☐	☐

Thank you for participating in this survey!

System Number: __ __ __ Team Number: __ __ __ Member Number: __ __ __

Date: __ /__/ __ Cycle: 0

System Number: ___ ___ ___ Team Number: ___ ___ ___ Member Number: ___ ___ ___

Date: ___ /___/ ___ Cycle: 0

Implementation Team Semi-structured Interview Guide: SCIP Phase 1

INTRODUCTION:

Thank you for taking the time to talk with me today. As a member of the Statin Choice Implementation Team, you have special insight into the factors that impact the up-take and use of the Statin Choice Decision Aid in your health system. We are very interested in understanding your perspective.

In this interview, I will be asking you questions about your thoughts and experiences related to implementing the Statin Choice Decision Aid. I want to assure you that your participation is voluntary and that you can choose to stop at any time. If a question makes you uncomfortable or you do not want to answer, that is fine; you do not have to. To respect your privacy, all of your responses will be held in confidence and no one will be able to connect you to any of the things you say.

You should feel open to express your thoughts and experiences honestly and to ask for clarification if any questions seem confusing.

Okay, do you have any questions before we get started?

WARM-UP CHAT (unstructured):

Okay, great. So, first I'd just like to learn a little bit about you. Please tell me about what you do in this health system. Why were you chosen to be on the implementation team? How long have you been here? Have you participated in any research studies before? Etc…

Transition: Okay. Thank you. This is all very helpful to me.

SDM CULTURE:

Every organization has a unique culture and way of doing things. I would like to hear your perspective on the culture of this health system. How would you describe the culture of this health system?

Probes:

- How is it different from other health systems?
- What are its major priorities?
- How does this health system work to make care more patient-centered?
- Have you ever heard of the concept of shared decision making?
- How does this health system work to encourage shared decision making?

Transition: I understand. This is very helpful.

System Number:___ ___ ___ Team Number:___ ___ ___ Member Number:___ ___ ___

Date:___/___/___ Cycle: Pre/Post

CAPACITY FOR CHANGE:

As a member of the Statin Choice Implementation Team, I know you're aware of the challenges involved in changing behaviors and routines. I am interested in hearing your perspective on this health system's potential for change. How would you describe the capacity of this health system to make system-wide changes in practice a reality?

Probes:

- Please describe an example of a time when this health system tried to make a change and succeeded
- Please describe an example of a time when this health system tried to make a change and failed
- Please describe your perception of the factors that are important in making change happen in this health system in general

Transition: I understand. This is excellent information and very helpful to us.

IMPLEMENTATION STRATEGY FOR SCDA:

For the rest of the interview, I'd like to focus specifically on your thoughts and experiences related to implementing the Statin Choice Decision Aid. Implementation strategies are any and all of the things we do to encourage a change in practice. Implementation strategies include things like training clinicians, changing processes or policies, or introducing reminders or incentives to change practice, etc. Implementation strategies can focus on the intervention, the clinician/practitioner, and/or the system/context. In your opinion, what strategy or strategies will be (or have been essential) to effectively implement the Statin Choice Decision Aid in this health system? In other words, if you could design the perfect implementation strategy for Statin Choice in this health system, what would it look like?

Probes:

- Describe your experience in working with the other implementation team members
- Where do you turn when you need support, help, or direction in achieving your goals?
- In what ways could our research team better support you?
- In what ways could implementation teams at other MCCN systems better support you?
- What has been your experience participating in research? Enjoy it? Is it a burden?
- How do you think the research activities impact the implementation efforts? Make it easier? Make it harder?

Transition: Okay. This is good for us to know.

This has all been very helpful. I think that's all I need unless you have any other comments or questions.

Okay, thank you again for taking the time to chat with me about this.

System Number:___ ___ ___ Team Number:___ ___ ___ Member Number:___ ___ ___

Date:___/___/___ Cycle: Pre/Post

Statin Choice Implementation Project: A Shared Decision Making Study of the MCCN
Implementation Team Semi-structured Interview Guide

System Number:___ ___ ___ Team Number:___ ___ ___ Member Number:___ ___ ___

Date:___/___/___ Cycle: Pre/Post

Implementation Team Focus Group Guide: SCIP Phase 1

INTRODUCTION:

Thank you for taking the time to participate in this discussion today. As members of the Statin Choice Implementation Team, you have special insight into the factors that impact the up-take and use of the Statin Choice Decision Aid in your health system. We are very interested in understanding your perspectives.

I will be facilitating the discussion. It is important to hear everyone's perspective and I encourage you all to share your thoughts, but to do so in an orderly fashion. To encourage and direct discussion, I will be asking you questions about your thoughts and experiences related to implementing the Statin Choice Decision Aid. My colleague will be observing the discussion and taking notes. The focus group will also be audio-recorded for analysis.

This is a research study. Your participation is voluntary and you can choose to stop or leave the room at any time. If a question makes you uncomfortable or you do not want to answer, that is fine; you do not have to. To respect your privacy, all of your responses will be held in confidence and your names will be deleted from the audio transcripts. No one will be able to connect you to any of the things you say.

You should feel open to express your thoughts and experiences honestly and to ask for clarification if any questions seem confusing.

Okay, do you have any questions before we get started?

WARM-UP CHAT (unstructured):

Okay, great. So, first let's just go around the room and do some quick introductions. Tell me your name, what you do in this health system, and what your role has been on the Implementation Team so far. If you are not sure or have not been actively involved to date, just say so; your perspective is still very important to us.

Transition: Okay. Thank you. This is all very helpful to me.

NPT REMINDER:

During our last visit, we did an assessment at the end of the workshop that was intended to help us understand important factors related to succeeding in this project. Some of you were probably there and might remember it. We had you answer a series of questions by raising and lowering your hand as we moved an indicator along a bar on the screen. Do any of you remember this?

Okay, well, today we are going to have a more in-depth discussion about some of these things. We hope it will help give all of us more insight about how to learn from and guide success here and in other organizations.

COHERENCE:

One of the things we know is that for new practices to be taken up, they need to make sense to those involved. The implication of this is that everyone involved must be informed about what the new practice is and what it means. This can be particularly confusing in a project like SCIP. How would you describe the overall goal of this project? How do you think others in this health system would describe the overall goal of this project?

One of the things we talked about in the last visit was making the distinction between shared decision making and the Statin Choice tool itself. How would you describe the challenge of making this distinction? Is there confusion or mixed messages? Within this group? Within the leadership? Among the clinicians?

This is a major implementation project that crosses the entire health system. Do you feel like you had a good sense of what you were getting into? Was the scope of the activities required a surprise? How so?

Every health system and organization has numerous competing priorities. Where does this organization find value in implementing the Statin Choice tool or shared decision making? Explain.

COGNITIVE PARTICIPATION:

In order for interventions to be taken up in practice, it inevitably requires people to get involved and to actively participate. What has the level of engagement been within this team? Are there individuals or groups that have been less or more engaged? Have people been added to or removed from the team? Why? What has the effect of this been? What about the level of engagement outside of this team?

Transition: Okay. This is good for us to know.

COLLECTIVE ACTION:

SCIP is a complex intervention, particularly because it requires participation from so many different individuals and departments. How have you organized your work? How have you allocated the various tasks required? How have you supported one another in accomplishing the various tasks? How have you held one another accountable?

REFLEXIVE MONITORING:

When we started this project, we spent some time brainstorming about how implementing Statin Choice would bring value to your organization. Have you thought more about this as a team? What about your individual work related to this project? Do you feel it is worthwhile? How do you go about knowing whether the intervention is bringing value to the organization? To you personally? Have you made modifications in how you are working as a team or as an individual? Why or why not?

System Number:___ ___ ___ Team Number:___ ___ ___

Date:___/___/___ Cycle: Pre/Post

This has all been very helpful. I think that's all I need unless you have any other comments or questions.

Okay, thank you again for taking the time to chat with me about this.

Leadership Receptivity Survey

The Statin Choice Implementation Project

Your health system is participating in a research study of the Mayo Clinic Care Network. The purpose of this questionnaire is to obtain information from you about your organization's priorities and needs and your experiences in trying to implement the Statin Choice Decision Aid into routine primary care practice.

This is a research study and your participation is completely voluntary. To respect your privacy, this survey will be de-identified as soon as you return it. No one will be able to connect you to your responses. By completing this survey you agree to participate in this research study.

Your answers to these questions may help direct efforts to improve the quality of care for patients in your health system and generate research knowledge that may improve care for many others.

If you have any questions or comments about this survey or study, please call: 1-507-XXX-XXXX

Thank you for answering these questions!

© Mayo Clinic Care Network Learning and Research Collaborative

Some Questions About You:

1. What is your leadership role in your health system? [please ☑ one]

   ☐1 Health system CEO or equivalent
   ☐2 Director of primary care practice or equivalent
   ☐3 Director of information technology or equivalent
   ☐4 Director of quality improvement or equivalent
   ☐5 Other (specify) ______________________________

2. What is your gender?

   ☐0 Female
   ☐1 Male

3. What is your age?

   ______ years

4. What is your race? [please ☑ one or more]

   ☐1 White
   ☐2 Black or African American
   ☐3 Asian
   ☐4 Native Hawaiian or Other Pacific Islander
   ☐5 American Indian or Alaska Native
   ☐6 Other

5. Are you of Hispanic or Latino origin or descent?

   ☐0 Yes, Hispanic or Latino
   ☐1 No, not Hispanic or Latino

System Number: __ __ __ Leader Number: __ __ __

Date: __ /__/ __ Cycle: 0

Some Questions About Shared Decision Making:

Shared decision making is an approach to clinical decision making that patients and clinicians engage in together. Please state the extent to which you disagree or agree with the following statements. [please check ☑ one response for each statement]

6. In general, I believe that shared decision making…	Strongly Disagree	Disagree	Slightly Disagree	Slightly Agree	Agree	Strongly Agree
a. …improves patient satisfaction	☐	☐	☐	☐	☐	☐
b. …increases health care costs	☐	☐	☐	☐	☐	☐
c. …improves patient outcomes	☐	☐	☐	☐	☐	☐
d. …is promoted by my health system/organization	☐	☐	☐	☐	☐	☐
e. …makes practice less evidence-based	☐	☐	☐	☐	☐	☐
f. …is too rigid to be applied to most patients	☐	☐	☐	☐	☐	☐
g. …is the typical way I make decisions with patients	☐	☐	☐	☐	☐	☐
h. …is the typical way most clinicians make decisions with patients	☐	☐	☐	☐	☐	☐

System Number: __ __ __ Leader Number: __ __ __

Date: __ /__/ __ Cycle: 0

Please indicate the extent to which you agree or disagree with the following statements. [please check ☑ one]

1. Participating in healthcare research is a priority of this health system.

☐1 Strongly disagree
☐2 Moderately disagree
☐3 Slightly disagree
☐4 Slightly agree
☐5 Moderately agree
☐6 Strongly agree

2. Participating in healthcare research adds value for this health system.

☐1 Strongly disagree
☐2 Moderately disagree
☐3 Slightly disagree
☐4 Slightly agree
☐5 Moderately agree
☐6 Strongly agree

3. Engaging patients in shared decision making is a priority of this health system.

☐1 Strongly disagree
☐2 Moderately disagree
☐3 Slightly disagree
☐4 Slightly agree
☐5 Moderately agree
☐6 Strongly agree

4. Implementing the Statin Choice Decision Aid is a priority of this health system.

☐1 Strongly disagree
☐2 Moderately disagree
☐3 Slightly disagree
☐4 Slightly agree
☐5 Moderately agree
☐6 Strongly agree

5. When this health system decides that change is needed, it is effective at making it happen.

☐1 Strongly disagree
☐2 Moderately disagree
☐3 Slightly disagree
☐4 Slightly agree
☐5 Moderately agree
☐6 Strongly agree

6. This health system has the necessary resources to succeed in implementing the Statin Choice Decision Aid.

☐1 Strongly disagree
☐2 Moderately disagree
☐3 Slightly disagree
☐4 Slightly agree
☐5 Moderately agree
☐6 Strongly agree

System Number: __ __ __ Leader Number: __ __ __

Date: __ /__/ __ Cycle: 0

7. This health system has sufficient support from the Mayo Clinic research team to succeed in implementing the Statin Choice Decision Aid.

☐1 Strongly disagree
☐2 Moderately disagree
☐3 Slightly disagree
☐4 Slightly agree
☐5 Moderately agree
☐6 Strongly agree

8. Collaborations with other health systems in the Mayo Clinic Care Network will help us succeed in implementing the Statin Choice Decision Aid.

☐1 Strongly disagree
☐2 Moderately disagree
☐3 Slightly disagree
☐4 Slightly agree
☐5 Moderately agree
☐6 Strongly agree

9. I am confident that our implementation team will succeed in implementing the Statin Choice Decision Aid.

☐1 Strongly disagree
☐2 Moderately disagree
☐3 Slightly disagree
☐4 Slightly agree
☐5 Moderately agree
☐6 Strongly agree

10. I believe that now is the right time to implement the Statin Choice Decision Aid.

☐1 Strongly disagree
☐2 Moderately disagree
☐3 Slightly disagree
☐4 Slightly agree
☐5 Moderately agree
☐6 Strongly agree

11. I believe implementing the Statin Choice Decision Aid will add value for this healthcare system.

☐1 Strongly disagree
☐2 Moderately disagree
☐3 Slightly disagree
☐4 Slightly agree
☐5 Moderately agree
☐6 Strongly agree

12. I believe that ensuring the success of this project is a worthwhile allocation of resources.

☐1 Strongly disagree
☐2 Moderately disagree
☐3 Slightly disagree
☐4 Slightly agree
☐5 Moderately agree
☐6 Strongly agree

Thank you for participating in this survey!

System Number: __ __ __ Leader Number: __ __ __

Date: __ /__/ __ Cycle: 0

System Leadership Semi-structured Interview Guide: SCIP Phase 1

INTRODUCTION:

Thank you for taking the time to talk with me today. As a leader in this health system, you have special insight into the factors that impact the up-take and use of the Statin Choice Decision Aid here. We are very interested in understanding your perspective.

In this interview, I will be asking you questions about your thoughts and experiences related to implementing the Statin Choice Decision Aid. I want to assure you that your participation is voluntary and that you can choose to stop at any time. If a question makes you uncomfortable or you do not want to answer, that is fine; you do not have to. To respect your privacy, all of your responses will be held in confidence and no one will be able to connect you to any of the things you say.

You should feel open to express your thoughts and experiences honestly and to ask for clarification if any questions seem confusing.

Okay, do you have any questions before we get started?

WARM-UP CHAT (unstructured):

Okay, great. So, first I'd just like to learn a little bit about you. Please tell me about what you do in this health system. How long have you been here? Have you participated in any research studies before? How familiar are you with this study? Etc…

Transition: Okay. Thank you. This is all very helpful to me.

SDM CULTURE:

Every organization has a unique culture and way of doing things. I would like to hear your perspective on the culture of this health system. How would you describe the culture of this health system?

Probes:

- How is it different from other health systems? What is its "identity?"
- What are its major priorities?
- How does this health system work to make care more patient-centered?
- Where does shared decision making fit among the priorities of this health system?
- How does this health system work to encourage shared decision making?

Transition: I understand. This is very helpful.

CAPACITY FOR CHANGE:

As a leader in this health system, I know you're aware of the challenges involved in motivating an organization to change behaviors and routines. I am interested in hearing your perspective on this health system's potential for change. How would you describe the capacity of this health system to make system-wide changes in practice a reality?

Probes:

- Please describe an example of a time when this health system tried to make a change and succeeded
- Please describe an example of a time when this health system tried to make a change and failed
- Please describe your perception of the factors that are important in making change happen in this health system in general

Transition: I understand. This is excellent information and very helpful to us.

IMPLEMENTATION STRATEGY FOR SCDA:

For the rest of the interview, I'd like to focus specifically on your thoughts and experiences related to implementing the Statin Choice Decision Aid. I am particularly interested in understanding how the tool fits with the needs and goals of your health system. In your opinion, why is this health system interested in implementing the Statin Choice Decision Aid?

Probes:

- How will implementing the Statin Choice Decision Aid add value for your health system?
- In your opinion, what are the biggest challenges to implementing the Statin Choice Decision Aid?
- In your opinion, what role does participation in research play in meeting the needs of your health system?
- In your opinion, what role does collaboration with other MCCN health systems play in helping your health system implement the Statin Choice Decision Aid?

Transition: Okay. This is good for us to know.

This has all been very helpful. I think that's all I need unless you have any other comments or questions.

Okay, thank you again for taking the time to chat with me about this.
