# Supplementary material for: Developing a toolkit to implement the Statin Choice Conversation Aid at scale: application of a work reduction model
Source: BMC Health Serv Res. 2019 Apr 24;19:249. doi: 10.1186/s12913-019-4055-8 (PMC6480421; doi:10.1186/s12913-019-4055-8)

Variable	System 1	System 2	System 3
Clinicians Sampled	50 (62.5%)	43 (50.0%)	13 (50.0%)
Age Mean	47.5	44.7	44.1
Age Median	46	42	41
% MD/DO	84	64.3	76.9
% NP/PA	16	33.3	23.1
% Female	42	53.7	53.8
% Hispanic	6	2.6	0
% White	77.6	82.9	100
% Black	2.0	0	0
% Asian	14.3	4.9	0
% American Indian	0	0.0	0
% Other	6.1	7.3	0
Patients Sampled	1376	465	186
Age Mean	53.0	64.2	55.2
Age Median	55.0	65.0	60
% Female	65.4	60.9	67.6
% Hispanic	3.5	2.6	2.2
% White	91.0	96.8	96.7
% Black	0.8	2.3	0.0
% Asian	0.4	0.2	0.0
% American Indian	3.3	0.5	1.6
% Other	1.3	0.2	1.6

Clinician and Patient Demographics

SCIP Phase 1

We went to 3 health systems to assess factors affecting the reliable and sustainable implementation of a SDM intervention for initiating statin therapy.

SDM and SCDA beliefs did not change significantly over study.

	System 1	System 2	System 3
Baseline Pt Involve	~75%	~78%	~85%
Baseline Champion	-	-	+
Proximal Diffusion	-	-	+
Distal Implementation	+	+	-
Distal Visits/Demo	-	+	-
Base Clin No Exposure/Routine Use	47%/16%	28%/16%	23%/23%
6 mo Clin No Exposure/Routine Use	21%/18%	24%/38%	0%/69%
Usage-Base (+/- EMR)	0(-)	0.1(-)	0(-)
Usage-3 mo (+/- EMR)	0.1(-)	1.1(-/+)	10.6(-)
Usage-6 mo (+/- EMR)	1.1(+)	2.9(+)	5.2(-)
Usage-9 mo (+/- EMR)	1.2(+)	1.5(+)	3.5(-)
Usage-12 mo (+/- EMR)	0.5(+)	2.2(+)	2.7(-)

A culture of patient involvement facilitates rapid uptake and passive saturation of usage in systems where clinicians are proximal and interact frequently. It is unclear if usage is maintained without EMR integration.

In larger, geographically disconnected systems, efforts must be made to demonstrate the tool to all clinicians, regardless of EMR integration. It is challenging to make all clinicians aware of tool in large, geographically disconnected systems.

System 1 Inner Setting

	System 1 #6	System 1 #7	System 1 #8	System 1 #9	System 1 #10	System 1 #11
Inner Setting						
Structural Characteristics	The outpatient setting is mostly primary care, but because we are at a satellite or smaller clinic and we don’t have a lot of specialty, I can occasionally consult for family practitioners for specific non-primary care issues			I mean, we—"Well, this is the way we do it." I think in the last eight years, there's been a huge cultural change here with regards to us working together as a team collaboratively, working on how to solve the problems for the patients. By a team I don't mean just physicians, I mean nurses, administrators, everybody. I think it's gotten a lot better since then. We still have groups of	Well, I think a huge thing is that we are physician-led. The position of president which is a physician, and CEO, are co-equals. If there’s a difference of opinion the physician prevails and the CEO can take it to the board of directors.	t’s fairly obvious through—and I imagine, taking our previous conversation—just our governance is really built in such fashion that stimulates shared decision making. It’s not, I say something and it goes, but it’s a rather, very, very engaging culture that is trying to boost that.

				doctors who like to be independent and don't like to be told.		
Networks and Communication	I mean, because we don’t work directly with System 1 Main, we mostly do our own little thing in Crookston under the supervision and guidance of Place. It’s a little different.<br><br>I think they have focused on that primarily, so I can’t even think of any system-based changes that have occurred. Now, being from Crookston may	We’re really at well somewhat the beginning of that process. Where in the last year we’ve worked hard on alignment of our goals and key objectives as a health system. I think now probably for the first time in our history we have a really strong cascading of those all the way down to few top-level objectives that we have all the way down in the organization to		I think we still struggle a little bit with that, but it's gotten a lot better. I mean, I think for the longest time—not the longest time, but after we integrated in 1997, I think for the next five to eight years, we acted still as doctors outside of the system.	We will have a leader on this project and I think we’ve had heavy involvement with different things we’ve done with EMR, and there’s been really good physician leadership. I think it comes back to if you get the right physician leader it’ll work. If you don’t, it won’t work. It’s that basic here. You can’t drive it from the	Let’s say, if you have a deal with—if you were facing some bigger challenge, let’s say, with Epic, literally, it was only a phone call away, let’s say, for me to get a hold of System 1’s president or a CEO or anyone from a C-suite or literally anyone from Physicians’ Leadership. Just being able to bring these things into a proper perspective and discuss them at such a high level when we need support or something.<br><br>Also, I think we do a fairly good job with communication and transparency. There aren’t too many [inaudible 14:11] year round, and we clearly state our intentions. We share plans ahead of the time. It’s never done in some, “This is it, live with it,” but we’re really trying to be as engaging as possible, so that we can incorporate multiple views and whatnot, and people know

	be the limiting factor.	the front lines. That’s good<br><br>With a tool like this not only do we want to deploy a tool like this so that it can help some people. Actually, I’m very interested in where we have tools deploying them well. Doing it as a series of standards so if a thing is useful for a patient, we want that to be available to them 100 percent of the time without fail. It shouldn’t matter in our organization what door you come in, what physician you			administrative side.<br><br>I mean have there been some failures? Yes. Why’d it fail? Because the physician person wasn’t strong enough or we either elected the wrong person or made the project too big which can make it fail or didn’t understand how we wanted to involve the other physicians.	that. Therefore, they feel that change wasn’t necessarily just pushed down their throats, but rather, that they were contributing a part in that process of change.

		see, or what service you interact with first that those tools are available to them.<br><br>Organic, we're good. Process we're not as good, but we're gonna get good.<br><br>For instance, we had a series of recent employee forums and my partner, our president and myself just spent about three weeks traveling around the whole health system talking about world-class care. One of the center pieces of that we actually used storytelling.				
Culture	Okay. It is definitely patient-centered more	We really do try to organize ourselves around the	I think the culture is our health system listens to the	I think the culture here is one of putting the patient in	The culture is just do what's best for the patient and	Well, generally, I'd say very friendly, receptive, open, generally, to new things.

	than anything.<br>Interviewer: Okay.<br>Interviewee: I think we—at least compared to previous places that I've worked with—we really do give that extra step to get the community a bit more involved in their own health care. Because a lot of the leadership is still in the medical field, they can do that along with keeping the patient in the primary focus.<br>I think overall that is the culture<br>However, in Crookston—again, things	patient. The needs of the patient do come first here.<br><br>I'd say a relatively flat feeling organization that most things that we get done we get done around here though collaboration and team work. Not too much through the hierarchal structure because we think that interferes with improvement activities.<br><br>I think now probably for the first time in our history we have a really strong	end user. I think a lot of the ideas are generated from us, as clinicians, and implemented in a down-up approach rather than just coming down from administration that you have to do this for a lot of projects and patient care-type stuff.	the center, the patient and their family, I think. I'm not talking just physician wide, I'm talking the system wide. I think they—everybody wants to put the patients' best interests first.<br>so I think our culture is one of community-based, not profit, patient-centered and looking at what's best, not only for the patient, but for the community.<br>We've started getting a little bit more into community wellness and those kinds of	things will work out, and work as a team. It'd be teamwork and do what's best for the patient.<br><br>we've always said that for any project to succeed, you have to have a physician leader. If you don't have a physician leader it isn't gonna fly.<br><br>We will have a leader on this project and I think we've had heavy involvement with different things we've done with Epic, and there's been	I think it's very, very patient-centered and really, the basic philosophy is that we're all here to provide just the best care we can for our patient.<br><br>I'd say there is definitely a fairly open-minded attitude toward accepting patient as an actual active member in the process of care.

	are different in our smaller community—we were more interested in a different candidate that they did not agree with at all. We made our case behind why we wanted him, and ultimately, he was chosen and he's our new clinic manager. I think this is probably one of those bigger times where leadership really listened to those who are over there in the front lines, like you say<br>Yes. The leaders in this organization listen to those	cascading of those all the way down to few top-level objectives that we have all the way down in the organization to the front lines. That's good<br><br>Organic, we're good. Process we're not as good, but we're gonna get good.		things the last five to ten years.<br><br>and so we have a lot of protocols that in PEDS works very well, because we've all agreed this is the way we're gonna do it, even though I'm on the winning side, and sometimes I'm on the losing side. When we vote, we just—this is the way we do it. Interviewer: Mm-hmm. Interviewee: I'm seeing that more system wide—Interviewer: Mm-hmm. Interviewee: -over the last several years,	really good physician leadership. I think it comes back to if you get the right physician leader it'll work. If you don't, it won't work. It's that basic here. You can't drive it from the administrative side.	

	who work for them. It is a very exciting type of culture for me. It seems that they are updating us on what health care is nowadays and where it’s going to be, and then they allow us to decide how to get there with their guidance. Then they help things happen.			as to people saying, "This is the way we should be caring for this disease. This is how we should take care of Diabetes. This is what we should do for monitoring the Diabetes. These are the protocols." To be honest, some of it is being forced upon us by—<br><br>I think it'll be beneficial. I think if I wanted to say there are any land mines, it's gonna be the Family Medicine Department.<br>Interviewer: Mm-hmm.<br>Interviewee:		

				They're a great group of doctors and providers, but they challenge you. Interviewer: Mm-hmm. Interviewee: Which I think is good. I mean, they'll say, "Well, why are you implementing this?" They'll ask the whys and all that stuff, which I think is good, because if we can't explain that, then maybe they're right.		
Implementation Climate		I think that isn’t because of the recalcitrance of people as much as it is not a strong enough		I mean on a scale from one to ten, we're probably—we're not at a ten. It's not like implementing		

		process for implementation of those things that I talked about with high reliability. We clearly have not been as strong. We've been strong in the organic part of doing things right. We have not been as strong in the process part of doing things, right?		will be, boom, just like that. I think we're probably at a seven or something like that.		
-tension for change	Interviewee: We recently, I think this year or earlier this year, we got the CV wizard. Interviewer: Okay. Interviewee: Basically what that's doing is also providing risk for cardiovascular	I think that it's more been informal in the beginning. Again with our more formal work that we're starting on right now with value management.<br><br>Today if you come back a	I think a lot of people overuse statins in our organization. Maybe that was what this initiative came from. For primary prevention, I think it'll be helpful from pushing	Great. Where would you say shared decision making fits among the system's priorities? Interviewee: I think it's high. I don't think it's the highest, I think it's high. I think—it's interesting. I		

	disease over ten years assessment based on patients’ lab work and comorbidities. We started implementing that. It has been not as impressive as I would have hoped.	year from now, I think you’ll see it more formally. I do think that you’ll get the idea that people want to do that here. That they do. It is in their mental check list.<br><br>Oh, yeah. I can think of lots of examples where we were I’ll say less successful than we wanted to be. I can use as an example the improvement of some of our quality measures.<br><br>While I firmly believe that equality of care here is very good. You	patient quality.<br><br>My only question is the CV Wizard. If that provides enough—if there’s not as much additional benefit with this—I mean, if the numbers are matching up the same and there’s not additional education material that’s really helpful with it, then to go to two different places, from an efficiency standpoint, I could see that being a barrier.	think I see the physicians pushing that a lot more than anybody else, to be perfectly honest. Examples in pediatrics are talking to the families, "Well, do you really need an antibiotic or not?" Giving them some of the facts about ear infections and going away on their own—		

		have to really prove that on a day to day basis by performance on quality measures. We have been slower at being successful in those than I want to be				
-compatibility	Our community is much smaller. I think that allows us to be more personal with the patients that we care for, cuz we know them from the community itself. I think the latest thing that we have implemented that's been getting the community a bit more	You're gonna hear as you visit around that we're focused on developing ourselves so that we deliver world-class care to the patients that we serve, and that is not a slogan. It's not a brag. It really is a measurable thing. In very area in our health system we're really	We have our—a separate—you've maybe seen something called the CV Wizard. I don't know if anybody's mentioned that or shown you that, but yeah. Tools to try to put the patient and their education at the front of that decision-	We're involving them more in the decision making. Again, that's not something that's unique to Altru. That's country wide as far as getting patients more involved in their care.	Well, we have a major three-year project right now on quality and service. It's being done by a group called Studer. You may have heard of them. The endpoint within that is called World Class Care	Absolutely. Again, there are some strides that we're making even by introduction of patient portals. We're trying to facilitate better patient engagement, so that patient is not just a passive part in the process, but rather, an educated component.<br><br>Again, things that we did with that collaboration with HealthPartners, with Cardiovascular Wizard—again, even though, as we all agreed, it's largely anecdotal data, still, reactions, as much as we can assess them, are fantastic from both patients and physicians. I'd say there is definitely a fairly open-minded attitude toward

	involved in their own care is the medical home approach.<br><br>It’s more of an individualized basis, allowing more responsibility to fall on the patients and just teaching them more instead of just orders and orders without reason<br><br>It is still difficult to have the patient understand the severity of a ten percent risk of cardiovascular event over ten years. It is difficult. They feel, oh, ten	setting goals on world-class levels of performance, world-class outcomes.<br><br>How does Altru work to make care more patient-centered? You already talked that that’s one of your goals and your tenants. How are you going about doing that?<br>Interviewee: Again I would say that right now while we sort of naturally do that a lot, we’re trying to be more disciplined about it.<br><br>How does	making. I think they’ve tried to set up some tools to be able to do that.<br><br>How do you think the health system has worked to encourage shared decision-making? Have they been looking at that for clinicians?<br>Interviewee: I haven’t seen any significant initiatives towards that.			accepting patient as an actual active member in the process of care.

	percent's nothing. It's a little bit of that type of mentality that has been a bit of a barrier. It takes a little extra time to explain, and even then, we don't always get the, I guess, response that we're looking for.	shared-decision making fit within the priorities of the health system? Interviewee: That is very, very high. Our values are our patients, are quality, our team, and our community. We look at the care that comes to the patient side as being a partnership with the patient that we have. We're not here to make decisions for our patients or tell them what's going to happen to them or something like that. We engage our patients as a full partner in the care that				

		they have here. This is dead center in the middle of what we want to be doing.				
-relative priority	Reference 1: 1.52% coverage<br>Do you think that there are things that the health system is doing to encourage shared decision-making, and what are those? Interviewee: I don't know if anything is active in terms of things that the health system is doing.<br><br>I think over the past, I would say, year or	There's a lot of work we have yet to do. Actually the project that you're working on I think relates to one of the things that still mostly in the future for us.<br><br>With a tool like this not only do we want to deploy a tool like this so that it can help some people. Actually, I'm very interested in where we have tools deploying them well.	I think it's good, if it's something that makes care better or more efficient in delivery. All the things that I do or on the EMR and trying to develop processes and tools and stuff like that, the way people implement it—the way I get providers to use it will be if it's more efficient or it affects care.<br><br>- CV Wizard has been my	there's also other priorities that I worry about more than the average employee would about market share and expansion and those kinds of things. I think the other priority we have is as health care is changing, we have to redesign how we care for our patients.<br><br>Great. Where would you say shared decision making fits	That's a more difficult one. I think you'll have areas where people are committed to it based on how they were trained and where they were trained. It'd be hard for me to say we have an active program where we're doing the shared decision-making<br><br>I think we're so advanced in our IS that	Well, the big focus, obviously, is—and I'm not even sure if I can really separate this from patient-centered care, but I think we have a very, very strong focus on quality.<br>Okay, how do you think the health system is working to encourage shared decision making? Interviewee: Well, I think even us engaging in this effort is a sure sign of the readiness. I mean, I can't say that we have, necessarily, a long history of doing it, but definitely it's becoming more and more obvious.

	two, most of the focus in the goals, I guess, for the health care system has been mostly directed to getting the docs to adjust to the CMS guidelines and to the CAPS 11:44 survey situation.<br><br>Okay, and the CV wizard tool that you mentioned—you mentioned some paper tools of it. Is that integrated into your EMR? Is there a web-based facing of it? Interviewee: It is a web-based thing and it has an interface with the Epic system. You	 Of course, it starts with our strategic plan. We’re trying to streamline that process so we are working always on a limited number of key improvement efforts. That we mean to make meaningful progress on and not sort of dilute our attention 5,000 different ways.<br><br>How does shared-decision making fit within the priorities of the health system? Interviewee: That is very, very high. Our values are our patients, are	baby, and working with health partners in implementing it here, so I’ve rolled it out and trained everybody in primary care and all the rest. Very successful, because it affects patient care, and it brings information to the provider that they’re searching all over the place to find.<br><br>I’d have to see it. What I’m trying to picture is how would this work with the CV Wizard, is what I’m trying to	among the system's priorities? Interviewee: I think it's high. I don't think it's the highest, I think it's high. I think—it's interesting. I think I see the physicians pushing that a lot more than anybody else, to be perfectly honest.<br><br>We've also gotten more into wellness, and we've invested money in local YMCA. We have what's called, "Choice Fitness," which we put a lot of money into and to empower people on their own.	this is just kind of a fun little challenge to see how we can do it. Now one of our objectives going into it was to convince Mayo that they should go with Epic and then last month [Name] told me that Mayo selected Epic. That’s good.<br><br>We’ll succeed. I’m sure there’ll be bumps in the road. I think sometimes people have to, from both sides, respect that we got a lot of things	

	click on their little link thing in the Epic, and it uploads online. Then it prints out the two sheets, one for me, one for the patient. Then I'll use that.	quality, our team, and our community. We look at the care that comes to the patient side as being a partnership with the patient that we have. We're not here to make decisions for our patients or tell them what's going to happen to them or something like that. We engage our patients as a full partner in the care that they have here. This is dead center in the middle of what we want to be doing.	picture. I don't know for sure.	Why do you think that the System 1 is interested in implementing Statin Choice Decision Aid? Interviewee: I think it's back to our primary focus, what's best for the patient?<br><br>I think the biggest challenge for all these is skepticism of physicians, and implementing a lot of things. You'll have some that'll buy in right away. You'll have a large chunk that'll buy in over time, and then you'll have some that don't	going on. This isn't the only thing we're dealing with.<br><br>You know, people have to align their priorities so they know, yeah, we're gonna get this done, but what might be Priority A for someone at Mayo might be C here, just depending on the week you're in Mayo or whatever.	

				buy in.		
-organizational incentives					Now the difference between that project and the statin project is, (a) they’re not gonna get paid for the statin project and they got paid for the other one, and how long is it gonna take? Again, the statin project is a one-timer and if they’ve already been on statins they’re eliminated I assume. It has to be someone who’s just newly going on a statin drug.	

-goals and feedback						
-learning environment		For instance, we had a series of recent employee forums and my partner, our president Dr. Eric Lunn, and myself just spent about three weeks traveling around the whole health system talking about world-class care. One of the center pieces of that we actually used storytelling.<br><br>Organic, we're good. Process we're not as good, but we're gonna get good.			Interviewee: I think probably the best thing will be to get them to tape Dr. Montori's 23:03 talk tomorrow. I've seen that talk. I don't know how he's changed it, but he's a colorful interactive person. Whatever he chooses to do will be just fine, and I'll talk with Rod and make sure they video it.	We went live April 1st of 2010. Right now, we are Stage 7, hospital and ambulatory, and among the most wide—and the reason why I mention that truly is, good portion of that really is due to the culture that again, is receptive. It's fairly open to the change. Not without challenges, but generally, it's open to the change.<br><br>It's one of those projects. It's one of those moments that just feels right. Really, a lot of good things that we have in any area of our lives today, there was some kind of a research that was preceding that. Interviewer: Yeah. Interviewee: Brought all the good stuff. Being part of something like that—and we all know, in the end, there is a person, patient, and we can make a positive change in their care and their lives. There probably isn't anything more rewarding. I guess that this is definitely, just feels right. It feels great just to participate.

Readiness for Implementation				Well, I think some of the work we've done with Diabetes, tried to get patients in more regularly, trying to monitor them more closely. I would say it's successful, it's not done. I would say—<br>Interviewer: Okay.<br>Interviewee: -our—we've shown nice steady improvement, but I think it's gonna take a lot longer to be able to do that.<br><br>within the last month. We've struggled with physicians reconciling meds and—		

				Interviewer: Mm-hmm. Interviewee: - we've tried several different ways to do it and so far we failed. Now we're on version four or five, I think. We're hoping this one is the charm. When I look back on the failures, a lot of it was I don't think we had enough input from nurses and physicians as to what are your issues? What are your problems? I think that's created failures		
-leadership engagement	I think ultimately, from other organizations	most things that we get done we get done around	How do you think the health system has worked to	think you've got the support of the top physicians, the	It'd be hard for me to say we have an active	Well, really, I mean, it's obviously a multi-faceted approach. The first is, just by the virtue of improving the culture

	that I worked with when the leaders tell them, “These are the goals. Meet them or there’s gonna be consequences,” into a culture where you feel like you’re driving change and they’re just helping you get to where you wanna go, that’s basically what tells me that they’re very open to change.	here though collaboration and team work. Not too much through the hierarchal structure because we think that interferes with improvement activities.<br><br>We really are working very hard right now to take a step forward in the way that we lead our organization and the hardwiring of good process in the organization.<br><br>There’s a lot of work we have yet to do. Actually the project that you’re working	encourage shared decision-making? Have they been looking at that for clinicians? Interviewee: I haven’t seen any significant initiatives towards that.	last President, who has a lot more respect in the community of health care, probably as I do right now since I'm a newbie. Although, I have a lot of respect. I mean, you've got Dr. Ryan and you've got myself, who are both on board with this. I think it'll be an interesting project that we go through.	program where we’re doing the shared decision-making	and starting with some simple things, kind of like [inaudible 03:31] the routine where you acknowledge and introduce yourself properly and everything, so you have good dialogue with patient, and really trying to hard-wire into the staff, through various engagements, and education, that patient indeed comes first.

		on I think relates to one of the things that still mostly in the future for us.				
-available resources	I think recently—well, yeah, over the past few months to few years, we've been really trying to expand the services that we provide. We're not quite there yet, but that has been a focus, I know, because again, small rural area. We don't get a lot of different specialties down here.				In some areas we're already there. I mean I could name probably eight areas right now. I mean we've just obtained HIMSS 7 in our information technology which puts us in the top three percent, two percent in the country because we've obtained it for all of our regional clinics and for the hospital. That's huge.	Can you think of any times, at the system level, that you tried to implement a change, and it didn't go so well or failed? Interviewee: Absolutely. It does happen, and unfortunately it's not a short list either. The thing is, if you set things right, we really look at occasional failures or challenges more as opportunities rather than anything cuz, if and when it happens, at least you'll learn what not to do.<br><br>There's really a good dialogue on all sides. I think we have a good degree of communication. I think we have all the right people involved. I think there is a—from our perspective, we really feel there is a great degree of support, and I think, literally, if we had to bounce some thoughts, or ask for some additional resources or opinions, it was always readily available. A lot of things that I

					That's one we're world class.<br><br>I think we're so advanced in our IS that this is just kind of a fun little challenge to see how we can do it.<br>I know at times when I've had discussions with [Name] that I'm sure he's been worried about, well, these guys I'm not sure they know what they're doing or they're not organized enough and da-da-da-da-da. Been there, done	would ordinarily like to see, they're really happening. I have to say, in that respect, I feel very comfortable.

					that. We’ve done so many things. The biggest piece is the IS connectivity and the ability to transport information back and forth. We’ve got the best.	
-access to knowledge	Are you familiar with the Statin Choice Decision Aid? Have you seen it yet or been able to use it? Interviewee: No.		Have you seen the tool yet? Interviewee: I have not.	I would look at that as far as changes in statins or changes in other things. Again, you can't just tell the physicians, "Well, yeah, ten nation experts said change this and use this drug." It's the why. Interviewer: Yeah. Interviewee: We've been taught to be		Also, I think we do a fairly good job with communication and transparency. There aren’t too many [inaudible 14:11] year round, and we clearly state our intentions. We share plans ahead of the time.<br><br>There’s really a good dialogue on all sides. I think we have a good degree of communication. I think we have all the right people involved.

				fiercely independent and good thinkers, so we don't just accept it. We wanna know the why.<br><br>I think that the best way you counter that is with education. It's showing them why this is gonna work. Interviewer: Mm-hmm. Interviewee: Now, if we can't—if the why is not good, it's gonna fail.		

System 1 Inner Setting

	System 2 #12	System 2 #13	System 2 #14	System 2 #15	System 2 #16	System 2 #17
Inner Setting						
Structural Characteristics	I don’t interact with a lot of other	We do a lot of efforts and programs for the				

	people, except at meetings, and those are—I don’t know. From top to bottom, I would say there’s a gap in-between. You’re talking top, like leadership people	community, but at the same time, I think we do sometimes get a bad rap or bad name, cuz we are the only game here in town— Interviewer: Mm-hmm. Interviewee: - as the famous quote is.<br><br>Well, the group that I'm involved in with the Internal Medicine, we're part of one of the ACO models. One of the ACO—				
Networks and Communication	I don’t interact with a lot of other people, except at meetings, and those are—I don’t	Mm-hmm. As a clinician on the front line, do you feel engaged with that—with this implementation and these	We—during our quarterly meetings and our grand rounds, we actually even also have a newsletter that		We talk about that in meetings and discussions, our monthly meetings and try to involve the patients in	Okay. Can you think of some factors that you might perceive as helping those things along? Interviewee: Communication.

	know. From top to bottom, I would say there’s a gap in-between. You’re talking top, like leadership people<br><br>In our clinic, I think right now there’s 10 providers maybe; 3 nurse practitioners and we have a doctor out on medical leave, so I think there might be 6 or 7 Drs. in there right now. We’re not all on the same floor, so I think that sometimes that hinders	changes happening? Interviewee: Absolutely. Interviewer: Okay. Interviewee: I might not know everything up front, but they're always—I believe that the lines of communication have really opened up here—	goes out, cuz we’re trying educate physicians around more standardization of practice and more patient input into the care they receive. We continue to try to educate.		decision making in that way.<br><br>Reference 2: 1.36% coverage Because we—in our group we have eight, nine providers, nurse practitioners and physicians—and we do monthly meetings, hard meetings and hurdles and all these things, we discuss these things and try to involve, yeah<br><br>First of all, the leadership definitely. The leadership has to make correct decisions. Interviewer:	

	our collaboration,				Mm-hmm. Interviewee: Then to know about the providers and workers which are high level, working people which are not so high a level, then to start improving them by different [inaudible 13:40] like counseling them and educating them and telling them what they need to improve on.	
Culture	I think they always say that they're open, but sometimes there's not always that opportunity to get right to	- personalized, very individual. The plan of care, I feel as if it's being more individualized, not trying to group a lump of people into one	he culture—when it comes to the physicians here at System 2, I think that we have a very engaged staff who really do	The culture is interesting. This Community is a relatively small community. When we look at consumer	The culture of System 2 basically is friendship. Interviewer: Okay. Interviewee: We do life care instead of just	I think they go out of their way to make their employees feel that they are important—That's good. Interviewee: - for the welfare of the organization. They may seem like small things, but it seemed to me a rather large thing. When I came here, they have a cafeteria that

	that person. You have to go through a chain. I think that sometimes that hinders providers—hinders providers' relationship to those that are on the high end of leadership, such as CEO, and then his assistants as well.<br><br>Actually, walking down the hallway, everyone is very friendly and welcoming even to patients. I've seen Drs. Stop and show patients	group or another. Slowing down the pace a little bit. I see us with patient care. I know just directly in my clinic, I've really extended out my visits, the time that I spend with my patients. I feel like we're very community-driven.<br><br>I just—I feel like the organization really tries to just find new and innovative ways to not only hold us accountable, but hold the patients accountable.	strive for excellence and the best patient care they can deliver.<br><br>Well, if you're looking for—shared decision making—if you're looking for what is the best care for the patient, okay, that's where shared decision making fits. We have to really shake off the old mantra of doctor knows best.<br><br>Health care had always—health care, as long as most of us that are actually talking today	perception, which weighs heavily on the culture of the organization,<br><br>In the last few years, we've gone through a real culture revolution and have implemented a high, medium, and low structure. With every leader, you're asked to evaluate all of your caregivers on performance. The highs are supposed to be recognized and rewarded. The mediums are supposed to be recognized and moved up. The lows are supposed to be recognized and	healthcare.<br><br>Well, definitely patient care is the main priority. Always they come first. Most of our work is based on what is good for them. On the same note, there's a lot of care about the providers, and we have done a lot of different improvements, especially after XXXX 03:43 has come here as our CEO	is set aside just for the employees. It's nice to go someplace where you're not—particularly when you're working with patients for 8 to 12 hours, to be able to get away from it for that 30 to 45 minutes.

	where they need to go, or at least walk them to the Courtesy Desk<br><br>Do you think as a clinician you'll be engaged in the process of sort of helping implement this, or?<br>Interviewee: No. [Laughter]<br>Interviewer: Do you perceive that that—?<br>Interviewee: No, I don't. Because probably what'll happen is they'll just come in and say, "Hey, the tool that		know it, has been a fee for service. Everything was paid for. Now they're going to have to share in the cost of that and certainly they need to share in that decision making and understand what are the benefits.<br><br>That's what we do. Educate.	either moved up or moved out. That's done a lot to improve the culture because everybody is kind of of this same mindset.<br><br>Again, because the community is such a small, close-knit community, whatever's happening with the community is affecting our caregivers.<br><br>Anyone who identifies as being a friend or a family member has a lower impression of us, which usually means our caregivers are going home and they're not		

	we showed you three months ago is now available. You can watch a tutorial.” That’s the way it’s gonna happen. Or, at our provider meeting that we have once a month in our group, someone may come and present it and say, “Hey, this is available.” That kind of thing. They’re not gonna ask me when and where. Not on that level, I guess.			able to defend what’s happening<br><br>As for culture, again, in my impression, it’s very driven by the community. We have a lot of caregivers that this is the only place they’ve ever worked. They don’t really understand the grass isn’t greener. They understand more about why aren’t you doing this? I’m entitled to this. I should be getting this.		

Implementation Climate						
-tension for change	I think you just have to keep up with the times that are changing. We do have a lot of older physicians and nurse practitioners, and patients as well that are reluctant to change.<br><br>Now I don’t know how many times a day I get asked by our patients, “Why did you change your name?” “What’s— Interviewer: “What’s up with that?” Interviewee: “What’s going on	We do a lot of efforts and programs for the community, but at the same time, I think we do sometimes get a bad rap or bad name, cuz we are the only game here in town—	Our identity—identity of System 2 to me is disruption of health care. How are we going to disrupt where health care is today?	We’re not always going to have 200 beds in this hospital. Understanding what that really means as an individual, I think, is maybe beginning, but it hasn’t permeated. I think that that’s what’s creating a lot of the fear, just uncertainty about health care in general.<br><br>It’s going to take a lot of time to get people to understand that their role is changing. This is a great test for those—when I look at		

	with that?” I say, “Well, we’re expanding. We’re changing, the culture is changing, the healthcare is changing. We need to be onboard with that.”			our cardiologists, I would say half are willing to go. When you look at our primary care providers, [laughs] I suspect they’re like, what are you talking about? You want me to do what? Again, they’re on an RVU 13:36 model.		
-compatibility	Yeah. Have you ever heard of the concept of shared decision-making? Interviewee: Yes. Interviewer: What does that mean to you? Interviewee:	The Portal is where we communicate back and forth with the patient, and though it has put some extra responsibility on me, I really try to answer each one of those individually. Then we can	What we want to do is, we want to be on the leading edge of that with, one, how do you standardize care where it can be standardized so that you provide the highest		Well, first of all, try to practice evidence-based medicine. Interviewer: Mm-hmm. Interviewee: Then, basically, trying to seek their input all the time. We are very good	

	It means that you’re gonna sit down with the patient and you’re gonna give them options and if it’s, “Hey, your A1C went up a point, what are we gonna do? In the next three months, are you gonna be able to cut back on your sugars or in your carbs? Or are you gonna be able to exercise three times a day or three times a week, to help bring that A1C back down? Or are we gonna need to adjust your medication	communicate back and forth.<br><br>I have been—or if you're talking about the shared decision making, I have been utilizing the Framingham—<br>Interviewer: Mm-hmm.<br>Interviewee: -model that we have on our computer. Its been very, very interesting, cuz I have a lot of 30 to 40 to 50-year olds that they take the statin.<br><br>It feels—I feel like it engages the patients to make decisions about their health care.<br>Interviewer: Mm-hmm.<br>Interviewee: It's	quality?<br>How do we make this work to make sure we can manage that population and give people who are seeking health care a lot of different alternatives in how they access that as opposed to the traditional setting?<br><br>The way we’re working to make care more patient-centered is around—we have initiatives around that, but it’s really—everybody’s doing it. We have a portal.		in providing them surveys for each visit and get their input, see what their satisfaction is in different kind of fields. Then we try to improve on them in which we are not meeting their expectations.	

	because you’re not definitely compliant with your lifestyle.<br><br>In my age group or younger. We don’t wanna wait two weeks for our appointment to get our results. I think that is patient-centered, but it’s also shared decision-making.	giving them that opportunity. It's giving them power. We're giving them the power back. Interviewer: Yeah. Interviewee: We're not dictating and telling them what they're supposed to be doing. It's a collaborative approach. It's a team approach—				
-relative priority	Do you think that the system is working to make things more patient-centered? Interviewee: Yes.	Yeah. Is there—and you said that's in your practice. Is the system doing anything to encourage that? Interviewee: I don't—I can't	The way we’re working to make care more patient-centered is around—we have initiatives around that, but it’s	Our priorities are to clearly get the clinics to grow in the northland. Our initial goal by June is 15 percent. Our goal	We have a very nicely worked new cafeteria, relaxing rooms, things like that. All in all, patient care the top priority, but also	What would you say are the major priorities of the health system? Interviewee: Delivering good care. Interviewer: Okay. Interviewee: Honesty. Actually being upfront and honest with the patient.

	Interviewer: How are they doing that? Interviewee: With the Portal and people. I don’t know how it’s gonna work out in the long run for the providers, because I get a few messages a week right now on the Portal.<br><br>last week, we have a provider meeting, of course, for our group, and talking about opening up our schedule to the public.<br><br>TeleMedicine	speak for that, but just—I mean from the very beginning when I started to practice, I really, like I said, tried to	really—everybody’s doing it. We have a portal.	organization-wide is to learn how to live within Medicare rates or break even with Medicare. Our third goal is the customer experience and constantly improving the customer experience. We talk a lot about being consumer or customer obsessed because we think that that’s the direction that health care is going to go.<br><br>What we’re learning is we created this life care experience and consumers aren’t really there yet. Consumers are	working with satisfaction for the providers.<br><br>Do you think that the health system is working to encourage shared decision making amongst the whole system with other clinicians? Interviewee: As far as we know in our group we definitely promote that. I have not enough knowledge for other groups, how they work, but I’m pretty sure the system as a whole promotes this, so I expect that everybody should be	

	is another avenue. I know at Work Comp Occupational Health, they've joined with Urgent Care so I've seen a lot of their interaction.<br><br>Do you think the health system is working to encourage shared decision-making?<br>Interviewee: Yes.<br>Interviewer: How?<br>Interviewee: Well, in the points that I've already discussed, with access to care,			still thinking about health care in terms of I'm sick; I'm going to my doctor. They're not thinking about their provider as being engaged in their health care. That's something—their health is outside of where their health care provider is. Our challenge is really how do you reach that kind of point where consumers are embracing that kind of message?<br><br>We know that consumers never want to be sick. They always want to	working on the same page, yeah.	

				be healthy. How do we help them achieve that goal? It really, truly is a matter of you really do almost have to change mindsets because consumers aren’t thinking of you in that role and providers haven’t thought of them in that role. Really focusing on that.<br><br>Cost. Again, one of our goals is to manage ourselves at Medicare rates. Every cost is scrutinized.		

-organizational incentives	We have to have so many people that are accessing this Portal that our communiqué with me via the Web and their email or whatever. Then I have to show I respond. They monitor that.					
-goals and feedback						
-learning environment	probably what'll happen is they'll just come in and say, "Hey, the tool that we showed you three months ago is now available. You can watch a		Reference 1: 3.19% coverage<br>When I talk about—when I say disruption, okay, the primary—one of the primary functions of Mosaic is to provide health and wellness to Northwest		We have worked in very different ways involving all aspects of care—as I said, hospital, clinic, home care, followed at home.<br><br>I think there are many, many things	

	tutorial." That's the way it's gonna happen. Or, at our provider meeting that we have once a month in our group, someone may come and present it and say, "Hey, this is available." That kind of thing. They're not gonna ask me when and where. Not on that level, I guess.		Missouri. That is our mission. Now through disruption, we all know in the changing arena of health care that we live in, continuing to do health care as we always have, i.e., more of a production model, high cost, quality variability has to change. What we want to do is, we want to be on the leading edge of that<br><br>We—during our quarterly meetings and our grand rounds, we actually even also have a newsletter that goes out, cuz		which I can mention in which we have worked as a team or individual and have shown good improvement.	

			we’re trying educate physicians around more standardization of practice and more patient input into the care they receive. We continue to try to educate.<br><br>for those that have been coming out now the last 5, 6, 10 years, they see a difference in the way medicine should be, and also in the way medicine should be for them. That’s what we do. Educate.			
Readiness for Implementation						
-leadership			I’ll be more		First of all, the	

engagement			than willing—I mean from my standpoint, I’ll be a champion from pushing that out to the docs. Dr. XXX will certainly be the physician lead on this. We would start with him. From what I see is if our EHR can make this an ease of use, I think the physicians, and APRNs, and PAs will gravitate to it. That’s where I really see is the tool’s out there. We want you to use it. I don’t see myself being a hammer or an edict on this to say, “Thou’lt		leadership definitely. The leadership has to make correct decisions. Interviewer: Mm-hmm. Interviewee: Then to know about the providers and workers which are high level, working people which are not so high a level,<br><br>Then, we basically were reviewing the emails and I had a couple of—I think three—meetings with our implementation team—two or three, I’m not exactly sure; talked to <Name> and <Name> off	

			shall." That's really not in my mind where we're headed. In my mind where we're headed is how do we put a tool out there that patients have access to and can understand, and physicians have access to that has ease of use, and can get the information to them that they want, so they'll want to use it?		and on. Basically, learning about the project to some extent. I was waiting for this visit of yours to basically get a good feel of it. That was my role, so I can say I have spent few hours and then—	
-available resources		I don't that that is an issue at all. That's what I see. If anything, there's always change going on here.	If you just look at the system, we have a lot of initiatives, in that we're on the cutting	Again, it depends who's involved in making the change. There are leaders in the	We have the necessary tools, and we have the willingness to do that. The team which is	I had not met Mickey before this project. I had not met Dr. XXXX, who just appears—I'm very impressed with. Interviewer: Yeah. How many times have you met with them, or have you met with all of

		Interviewer: Uh-huh. Interviewee: Sometimes it could become overwhelming— Interviewer: Yeah. Interviewee: - cuz you're trying to keep up with the change. Interviewer: Mm-hmm. Interviewee: Absolutely, there is capacity for change, and we show that every day in the things that we do.	edge with ACO-bundled payments, portal, moving to ICD-10. We are stretched pretty thin.<br><br>We already have several of the abilities and I do believe that if we can put the information in front of the providers, they will accept it<br><br>went smoother than I would've expected. I think the biggest issue that we did this well, is we had at-the-elbow resources. The super users who were at the provider's	organization that can just say, "This is what we're going to do." To some degree, it gets done. Other things are more difficult.<br><br>we didn't have anybody from the clinics—oops. That's wrong. We had people from the clinics on the team, but they were so busy they never came to the meetings. They were just not involved.<br><br>Again, it depends. Do you have the right leader? Do you have the right implementation	working is very much involved. Interviewer: Mm-hmm. Interviewee: They do regular meetings, so I see a good hope here as far as the willingness and the necessary involvement and the tools needed for the project.	them now? Interviewee: Yeah. We've met about three times. Interviewer: Okay. You at least know each other, when we start talking tomorrow. Interviewee: Yeah.

			elbow during that type of project.<br><br>Yeah. You've mentioned education is really important to getting a change—Interviewee: Mm-hmm. Education. At-the-elbow support.	team? Does everybody understand what you're really trying to do? Then things can move relatively quickly with this caveat. This organization is so thin in terms of resources.		
-access to knowledge	No, I don't. Because probably what'll happen is they'll just come in and say, "Hey, the tool that we showed you three months ago is now available. You can watch a	Have you heard of the concept of shared decision making? Interviewee: I cannot say that I have. I probably have—its probably been thrown out there, but no, I don't—I'm not familiar with it.<br><br>Is the system doing anything	I think the biggest issue that we did this well, is we had at-the-elbow resources. The super users who were at the provider's elbow during that type of project.		What does that mean to you, what you know of it? Interviewee: I really think whatever I know is that patients should be involved in decision making. Whenever we are proposing a plan or changing	A lot of information before changes are made. Explanations as to why changes are made. I think, if you're going to be successful in a project working out, you need a lot of communication in a way that people receive it, read it, or hear it.

	tutorial." That's the way it's gonna happen. Or, at our provider meeting that we have once a month in our group, someone may come and present it and say, "Hey, this is available." That kind of thing. They're not gonna ask me when and where. Not on that level, I guess.	to encourage that? Interviewee: I don't—I can't speak for that, but just—I mean from the very beginning when I started to practice, I really, like I said, tried to—			treatment or a new treatment or any tests, I think we should give them ample information and then involve them in decision making—what they think about this and what they wanna do.	

System 3 Inner Setting

	System 3 #18	System 3 #19	System 3 #20	System 3 #21	System 3 #22	System 3 #23
Inner Setting						
Structural Characteristics	Interviewee: This area here	During the last three-and-a-half	Yeah. We are a community-		I think it starts at the top from	

	I feel like it's very regional.<br><br>a lot of times they'll get care in those outlying clinics. Whether they be county or even tribal clinics and things like that, and then coordinating that with Kootenai.<br><br>At first, it was a little bit slow to get started, and a little bit awkward or cumbersome, because I would have two more people, and I'd have to get permission from the	years we have grown substantially. System 3 Medical Center, which was our prior name, had been a community hospital with very little involvement in a larger region and very little involvement with employed or affiliated physician groups<br><br>Reference 2: 1.20% coverage During the last three-and-a-half years we've moved substantially to becoming a regional medical center.<br><br>I think we had six physicians	owned hospital in a mid-sized resort community, which is an important attribute because it is a very cordial, friendly, service-oriented organization with many employees with long-standing tenure, and a real supportive environment.<br><br>We're really honored to be part of the Mayo Clinic Care Network, but we're also community-owned, and sometimes that smaller size can be disadvantageous. We don't have access to a big national system.		the board to XXX our CEO, who hired me, and to our executive team. They're very supportive of everything we're doing on the IT side. It's a partnership as opposed to an adversarial relationship.	

	patient. “Is this okay if they’re in here?” We always do. We always tell the patient this is a scribe.	who were employed at the time that I got here, and we have close to a hundred at this point.<br><br>It is the community's hospital. It is a quasi governmental institution. It's a hospital district essentially, you know, the county hospital for our County, and by extension for all of XXX.<br><br>We are kind of a weird combination of community hospital/regional medical center and county hospital. Using that—and I think with the				

		sense of community ownership and dedication to this hospital, we've been pretty successful, financially stable.				
Networks and Communication	I know myself, as a primary care, if I'm referring somebody somewhere I just pick up the phone and call the specialist.<br><br>We just go ahead and send the referrals through, but with our EMR at least, we can at least see things a little		The view, this view of learning, is that all the policies and procedures and guidelines in the world are not gonna be as valuable as educating our employees about our story and where we're going, and using a picture.	You know, I think people are attempting to provide coordinated care. However, I think that because there are so many different groups, there are specialists that don't necessarily belong to the same organization. I think that's one thing I have found, actually, quite difficult about starting		

	bit more readily. Not all of the clinics are on the same system, but we try to do as good a job as we can to not repeat things--			my practice here is just that the coordination of care is that much more difficult.<br>Here, you know, it seems like not everyone is on the same EMR, so I may not know when I see someone,		
Culture		Generally the culture of System 3 at the hospital, if I could take the physician culture first, derives a lot from the culture of XXX.<br><br>XXX is known, I think even nationally, for somewhat independent thinking. The	The other side of that is that healthcare has now become so very complicated, and there are so many rules and regulations that standardization of process and policies and procedures [inaudible 02:34] things becomes challenging	You know, I believe so. I mean, in terms of my new physician orientation, I think that there was a fair amount of emphasis on communication, communication skills, trying to elicit the patient's perspective, or, kind of, find	Having been at multiple different organizations, this is by far the best culture that I've been in.<br><br>I would compare what they were trying to get to to what we have here which is a wonderful	I would say that it is a—it's still a—there's a small-town feel within the hospital, and but the recognition that we're changing to something bigger.<br><br>To everything that we have today. Part of our culture is trying to hold onto that feeling of a smaller area, but also move us to where we need to go to survive, really, in this environment.<br><br>Right now, our culture—I would say there's a lot of

		sense of almost frontier independence is something that is really treasured here.<br><br>We have a large number of doctors who treasure their independence and struggle with some of the changes in healthcare that makes it less easy to be an independent physician.<br><br>That's one of the things that I think we have misperceived ourselves as being pretty patient centered. We need to recalibrate that perception.<br><br>The	because it's such a friendly culture. When you adopt mandatory rules, regulations, standards, guidelines, at times it can be a real challenge.<br><br>We were trying to find a way to do a better job of educating, training all of our staff and our physicians about our culture, and we came up with something called the XXX Health Way.<br><br>Organizational Development department come up with a new program called XXX Health Way, which is a training program	out more about that person as a "person," you know	culture. Very friendly. Very helpful. Very filled with integrity. Always trying to move forward.	change. I mean, it's definitely—we are a change culture, and I think most people are embracing that. They understand why, and where we need to get to be, but we're still—we like to think of ourselves as still very focused on safety and quality.

		independence thing is really important. The sense of questioning the validity of the information that's presented is also important—	for every single employee, all of our board members, all of our medical staff, on how we make decisions and work together as a team.<br>Then, really, the second, big-picture way of educating our organization on what’s most important is really the XXX Health Way program, and requiring a hundred percent of our employees to go through that training. We’re makin’ really good progress. Whenever you make somethin’ mandatory, it’s a challenge, but we’ve made the			

			training so much fun that employees really enjoy it			
Implementation Climate		Some of the physician-related stuff has been a real challenge, and it—we had issues with our infection rates in our operating room.			As you heard today, one of our major challenges is always dealing with the vendors. We constantly have to push to be able to make sure that they meet the timelines or they even give us timelines to where they can give us the development and be able to get us to where we want to go.	
-tension for change		I think that there's several different things that potentially could happen with this. One is				I think that when you ask anybody, they would say it is, but our numbers don't reflect that. It's, okay, what do we need to do? You can ask any employee—oh, yes, I

		it's too much of a hassle and people are moving too quickly and they just ignore it and they say, "I could give you this tool, but I think you probably should take this." And so they bypass the tool.<br><br>'ll give you a potential barrier. Electronic medical records. A pain in the ass.				care about patients, but our patients aren't saying that about us. We're doing a lot of initiatives right now on how do we change that.
-compatibility		Once again, I think because of our prior history of significant independence, there's a bit of paternalism that we have to get over.			What does that mean to you when you think about patients and clinician shared decision making? Interviewee: It has input from the patients primarily to try	I would say, as an organization, we've recognized that we need to improve in our patient engagement, and our—we have this new initiative going on called the XXX Help Way.

					to sum it up, and that would facilitate the communication requirements which was where IT fits in.	
-relative priority	Is there any initiative that you've heard of in this health system to incorporate shared decision making? Interviewee: I haven't been talked to about it.	What would you say the major priorities right now are for XXX? Interviewee: For us the biggest issue, really, we were trying to address is maintaining services in XXX.<br><br>Our intent is to become as technologically as well as socially—I'd say sophisticated once again. We use the word a lot. But it means that people don't	Like everybody else in healthcare, it's trying to navigate through the myriad rules and regulations associated with the Affordable Care Act, dealing with reimbursement decreases, extraordinary new requirements in information technology, value-based purchasing, population health, and doing all of that while still being	Do you think that XXX Health is working to encourage shared decision making? Interviewer: You know, I believe so. I mean, in terms of my new physician orientation, I think that there was a fair amount of emphasis on communication, communication skills, trying to elicit the patient's perspective, or,		Of course, they picked two that are doing better than us, and it was very eye-opening for a lot of people. I think there's really this buzz right now of, how do we engage our patients? How do we show—demonstrate this real commitment that each of us has, and make that a more apparent or transparent to our patients?<br><br>Can you give me an example of maybe one of those initiatives that you're working on to change patient engagement? Interviewee: Well, there's a few things, actually. We started, for the first time, a patient engagement committee, a steering committee.

		have to travel in order to get their healthcare.	a mid-size, community-owned hospital.<br><br>Shared decision-making particularly related to patients and clinicians sharing decisions together. Interviewee: Yeah. Well, I think we strive to do that every day. That fundamentally is part back to that culture of really a collaborative approach to patient care,<br><br>Probably, in some ways, no different than the other things I mentioned when I said that the education and training about	kind of, find out more about that person as a "person,"		

			this must be physician-led. It’s gotta be considered to be consistent with our values, which it would be. It’s gotta be fun.			
-organizational incentives						
-goals and feedback						Maybe we’ve lost some of our engagement with our patients along the way. For example, our patient experience scores aren’t very good right now, and that’s been—and part of the XXX Help Way, they—every single employee’s had to go through that, and they really showed us to our competitors. Here’s what they’re doing. Where do you think we are? Of course, they picked two that are doing better than us, and it was very eye-opening for a lot of people.<br><br>Anything else you would want in that implementation

						strategy? Interviewee: Certainly, our metrics, how we measure our success. What’s our baseline? What are we shooting for? That kind of thing. What our feedback plan is going to be to staff, to show that this was successful.
-learning environment		Our staff, because of the independence in the sense that nobody should tell you whether you have a flu shot or not, struggled with the sense that we were gonna move to a point where all of our staff, all of the consultants, everybody that comes into the hospital, mandatorily has a flu shot. It's just part of the rules.				

Readiness for Implementation		In general, implementation works when you have somebody who's a champion, who has enough of a clinical standing and enough involvement in, frankly, patients, that other people can see the change.	Well, our view as it relates to clinical programs, we always wanna have a physician champion. Physician leadership is just incredibly important to driving change,<br><br>Finding a physician champion and physician leaders to advocate for the change is hugely important.		Tremendous. Actually it's the most change that I've experienced, and I've experienced a lot.	What about any changes that have been tried as far as implementation goes that didn't go well? Can you think of any of those? Interviewee: Well, I think in the past, some of the initiatives that they've tried to do around our patient experience scores and things haven't been sustained.<br><br>I think a lot of those basic management type skills of really defining your scope, your charter, who's gonna—who your stakeholders are, your communication plan, what you're going to actually do, what's the life cycle, how you're gonna ensure—if we actually define all of those steps, then I think we'll be successful.
-leadership engagement		I think that's something we could do a lot better job in. Interviewer: Great. Interviewee: That's why I			Well, the strategy has to start with the requirements. What is needed in this project? I think we're gathering that.	t's run by someone from our C Suite, and it's even on the weekends. They come in on the weekends to run it, and it's just—many hospitals do a variation of it where everyone just goes around. Are there any pressing safety

		think the study is kind of compelling—Interviewer: Wonderful. Interviewee: -because it does engage people in that conversation. There's about ten conversations that are that way, some of which we really struggle with.			That strategy would be associated with that outcome or that goal of the project. It would be designed around it. As one of the gentlemen was saying today, we want this to lead to X, Y, and Z. That's really what I try to get that strategy aligned with for this project. Does this project meet those external goals as well as the internal goals of where we're trying to go to?	issues today kind of thing, and so many good things have come out of it. That has been—it was very well-planned, and it had real executive support, and everyone views that as a tremendous success.<br><br>Yeah, no, that's been very, very good. I think in terms of our culture, when an initiative has that level of support, and then really does the good planning work, that we can do—we can accomplish anything
-available resources	It happens, in some ways faster than others and in	ll give you a potential barrier. Electronic medical records.	I would say some of the bigger challenges		Yes, we have a lot going on. IT is one of those major	

	a lot of other ways, I lot slower than I would like. [Laughter] I guess it depends on the area.<br><br>We've done a lot with our EMR. There's a lot with our EMR that I've been begging, pleading for, and I feel like the wheels are very, very slow to turn. There are things that are redundancy issues. Technological issues. Things like that. Where it makes, obviously, our life more difficult--	A pain in the ass.<br><br>t's all beta. It's not anywhere near as good as Google even though it's billions of dollars spent on it. It hasn't fulfilled its initial promise. Fine. It slows down primary care physicians who need to do this as opposed to clicking, moving, and they need to talk to somebody and engage with them as opposed to clicking and moving. So we're actually instituting scribes—	we've had have been in the area of information technology. Yeah, but we kinda came up with a way. We created a computer lab training center here.		priorities, and we do have our ten or so goals that we work through—IT being one of them. That's primarily why I was hired here is because we needed to build a team—IT team—from the ground up.<br><br>That helped a lot, but again we're talking to a salesperson so we may need some help in the future with regards to escalation and your support, but I feel from the XXX health side, we can make anything happen that we have our	

	Well, I mean because obviously the EMR that we use is system-wide so, do I love it? No. It’s actually a very user unfriendly program. That’s something we struggle with a lot here.<br><br>It’s down often. Not completely, but it’ll be very slow. Those things, you know, oop, okay, let’s call IT. Nope. They don’t know why. Even on the weekends if I’m trying to do stuff.				control under. I think we’re okay there.	

-access to knowledge	Have you seen the Statin Choice Decision Aid tool at all? Interviewee: No.		There's gotta be value added for patients. How do we get that message out about, why this study—			Anything else you would want in that implementation strategy? Interviewee: Certainly, our metrics, how we measure our success. What's our baseline? What are we shooting for? That kind of thing. What our feedback plan is going to be to staff, to show that this was successful.

Of the 80 clinicians provided to the research team for participation in the research team, 50 clinicians responded to the baseline survey. The clinicians were surveyed at 3 time points: Baseline (Round 1), 6 months post baseline (Round 2) and 12 months post baseline (Round 3).

Table 1: System 1 Clinician Demographics from Round 1

	System 1 (N=50)
**What is your degree within your health care profession?**	
MD/DO	42 (84.0%)
PA	2 (4.0%)
NP	6 (12.0%)
Other	0 (0.0%)
**What is your gender?**	
Female	21 (42.0%)
Male	29 (58.0%)
**What is your age?-Years:**	
Mean (SD)	47.5 (11.8)
Median	46.0
Q1, Q3	37.0, 56.0
Range	(30.0-71.0)
**Race**	
Missing	1
Asian	7 (14.3%)
Black	1 (2.0%)
Native Hawaiian/Pacific Islander	1 (2.0%)
Other	2 (4.1%)
White	38 (77.6%)
**Are you of Hispanic or Latino origin or descent?**	
Yes	3 (6.0%)
No	47 (94.0%)

Response rates for clinicians:

Responded to all rounds	Round 1 only	Round 1 & 3	Round 2 & 3	Round 3 only
33 (41.3%)	9 (11.3%)	8 (10%)	11 (13.8%)	9 (11.3%)

Response rate by Round for clinicians:

Round 1	Round 2	Round 3
50 (62.5%)	44 (55%)	61 (76.3%)

Clinicians were asked on their knowledge of and use of the statin choice decision aid as well as a series of questions that assessed their beliefs on shared decision making (SDM Beliefs) and the Statin Choice Decision Aid (SCDA Beliefs). Higher scores on SDM beliefs (range 0 to 30) were consistent with generally positive beliefs and attitudes about SDM and its appropriateness in clinical practice. Higher scores on SCDA beliefs (range 0-18) were consistent with generally positive beliefs and attitudes about the Statin Choice Decision Aid and its appropriateness and usefulness in clinical practice.

Table 2: Adjusted rate of Clinician self-reported use of the SCDA.

	Round 1	Round 2	Round 3
Level of exposure of SCDA: Percentage of occasional or routine use (95% CI)	32.5 (25.6, 39.5)	59.2 (49.3, 69.1)	63.1 (54.3, 72.0)

Figure 1: Clinician's Beliefs about SDM by knowledge and use of SCDA in System 1

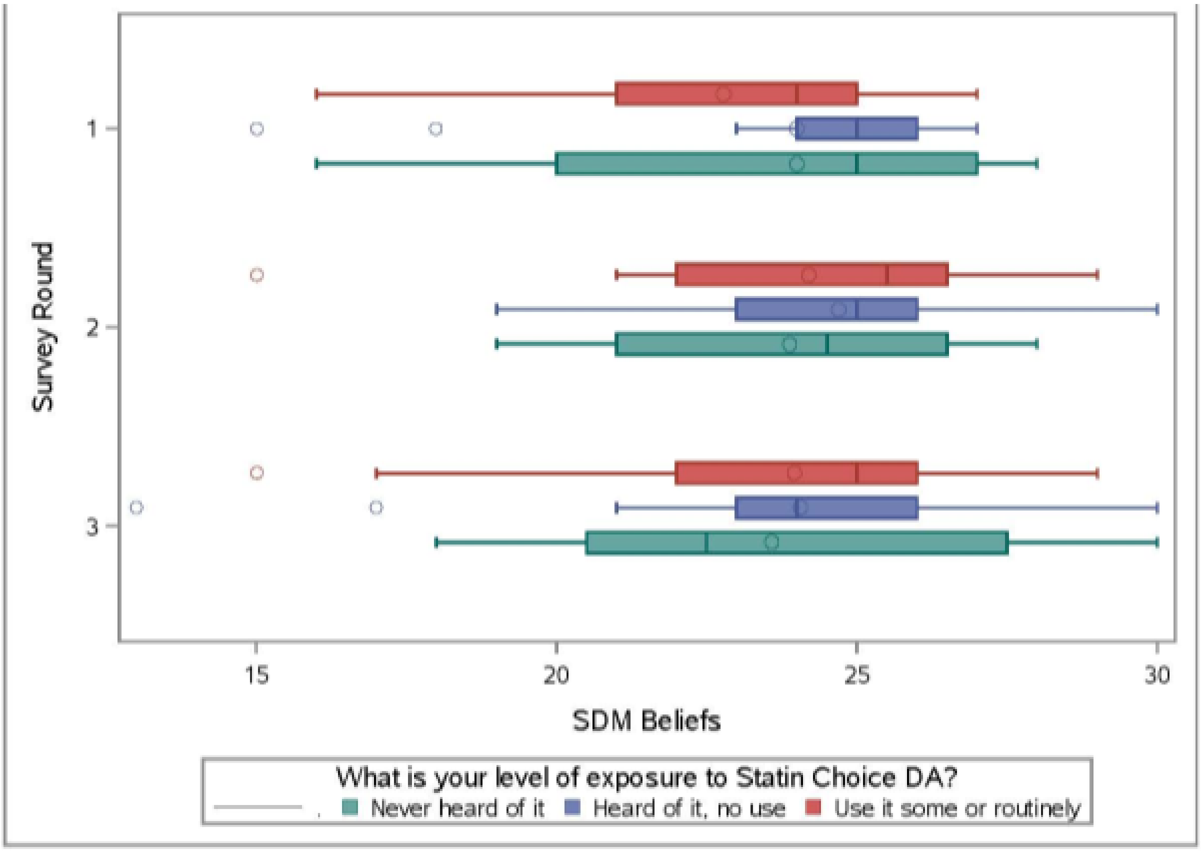

While the beliefs did not significantly differ by knowledge/use of the SCDA, over time we did see a shift of the beliefs. In Round 1 clinicians with use of SCDA had a lower level of SDM belief on average, by Round 2 this changed where clinicians with use had higher SDM beliefs than those without exposure to the tool and the average difference was even larger by Round 3. We hypothesize that the observed change occurred due to a move from one category to another

(never heard of it in round 1 to use it in round 2..), the SCDA having a positive impact on clinician's views of SDM, or (most likely) a combination of these factors.

Figure 2: Clinician's Beliefs about SCDA by knowledge and use of SCDA in System 1

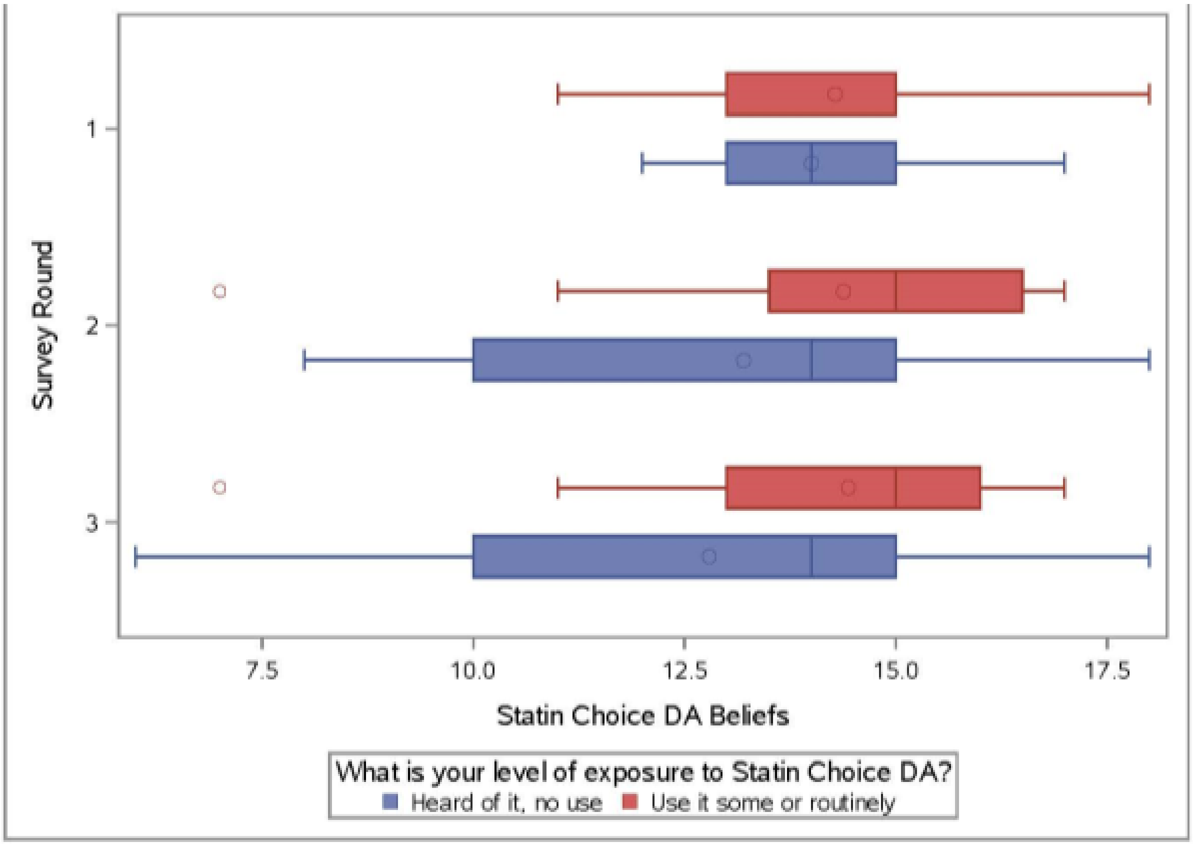

These questions were only asked of clinicians who reported at least hearing of SCDA (Round 1= 27 (26.5%), Round 2 = 29 (66%), Round 3=43 (70.5%)). For SCDA belief the average score increased per round with use and stayed higher than those who reported not using it.

Figure 3: Web reported usage rate over time in System 1

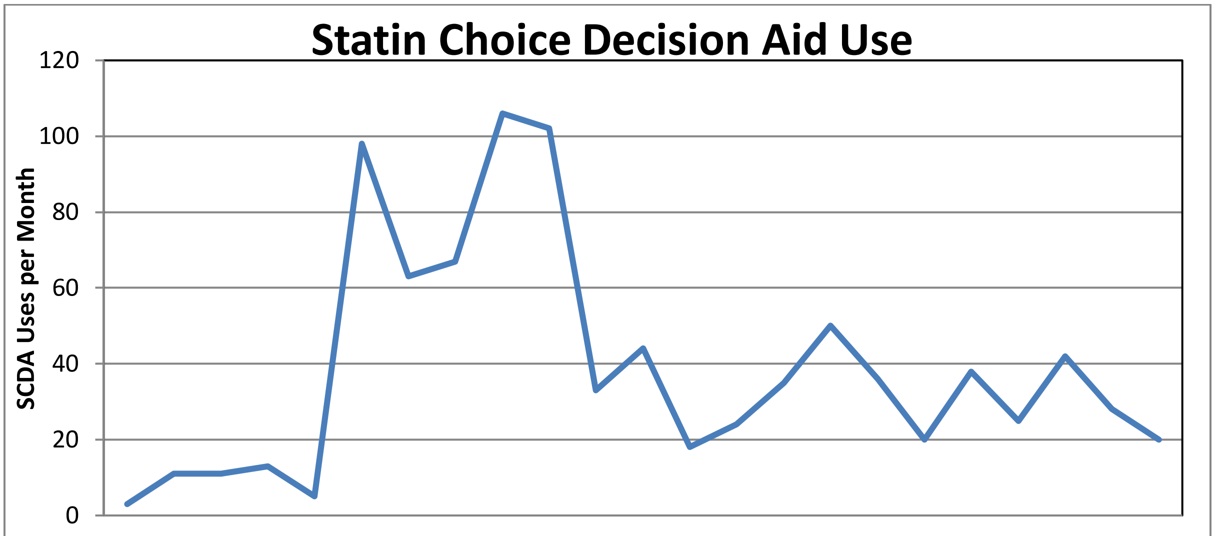

	Mar-15	Apr-15	May-15	Jun-15	Jul-15	Aug-15	Sep-15	Oct-15	Nov-15	Dec-15	Jan-16	Feb-16	Mar-16	Apr-16	May-16	Jun-16	Jul-16	Aug-16	Sep-16	Oct-16	Nov-16	Dec-16	Jan-17
System 1	3	11	11	13	5	98	63	67	106	102	33	44	18	24	35	50	36	20	38	25	42	28	20

Month/Year

Usage peaked around the fall of 2015 and declined to a steady use of 20-40 encounters per month.

Figure 4: Rate of usage over time of SCDA per clinician in System 1.

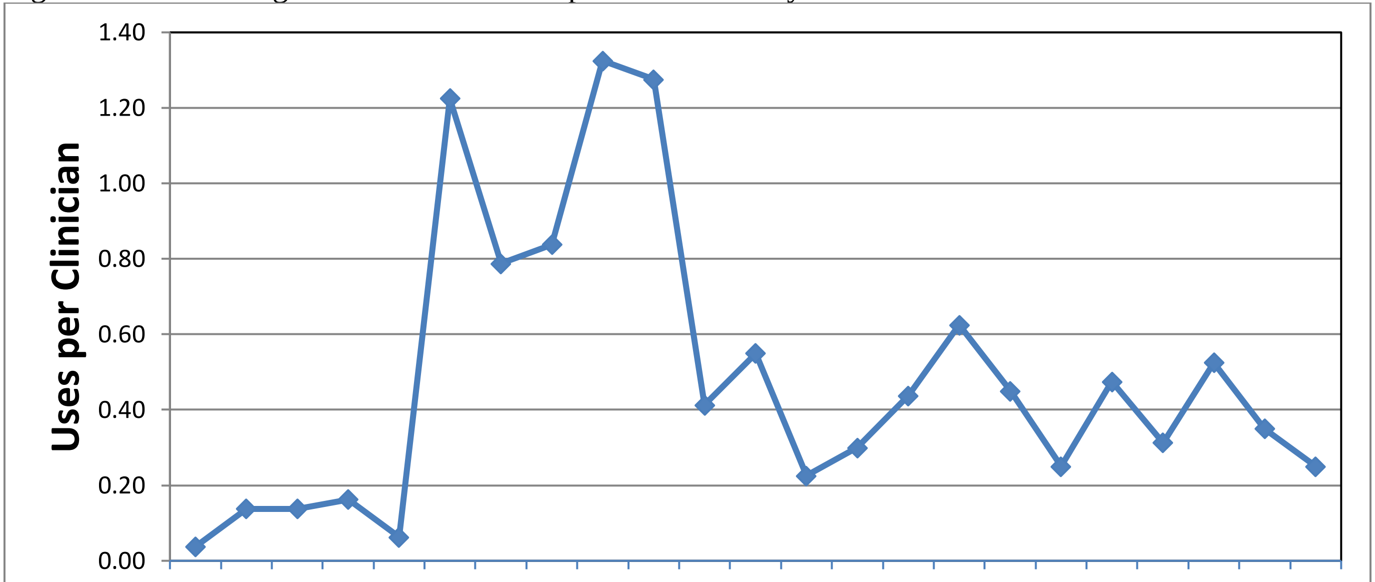

	Mar-15	Apr-15	May-15	Jun-15	Jul-15	Aug-15	Sep-15	Oct-15	Nov-15	Dec-15	Jan-16	Feb-16	Mar-16	Apr-16	May-16	Jun-16	Jul-16	Aug-16	Sep-16	Oct-16	Nov-16	Dec-16	Jan-17
System 1	0.04	0.14	0.14	0.16	0.06	1.23	0.79	0.84	1.33	1.28	0.41	0.55	0.23	0.30	0.44	0.63	0.45	0.25	0.48	0.31	0.53	0.35	0.25

Of the 86 clinicians provided to the research team for participation in the research team, 43 clinicians responded to the baseline survey. The clinicians were surveyed at 3 time points: Baseline (Round 1), 6 months post baseline (Round 2) and 12 months post baseline (Round 3).

Table 1: System 2 Clinician Demographics from Round 1

	System 2 (N=43)
**What is your degree within your health care profession?**	
Missing	1
MD/DO	27 (64.3%)
PA	3 (7.1%)
NP	11 (26.2%)
Other	1 (2.4%)
**What is your gender?**	
Missing	2
Female	22 (53.7%)
Male	19 (46.3%)
**What is your age?-Years:**	
N	40
Mean (SD)	44.7 (11.5)
Median	42.0
Q1, Q3	35.0, 53.0
Range	(29.0-71.0)
**Race**	
Missing	2
American Indian/Alaskan N	2 (4.9%)
Asian	2 (4.9%)
Other	3 (7.3%)
White	34 (82.9%)
**Are you of Hispanic or Latino origin or descent?**	
Missing	4
Yes	1 (2.6%)
No	38 (97.4%)

Response rates for clinicians:

Responded to all rounds	Round 1 only	Round 1 & 3	Round 2 & 3	Round 3 only
18 (20.9%)	14 (16.3%)	11 (12.8%)	11 (12.8%)	3 (3.5%)

Response rate by Round for clinicians:

Round 1	Round 2	Round 3
43 (50%)	29 (33.7%)	43 (50%)

Clinicians were asked on their knowledge of and use of the statin choice decision aid as well as a series of questions that assessed their beliefs on shared decision making (SDM Beliefs) and the Statin Choice Decision Aid (SCDA Beliefs). Higher scores on SDM beliefs (range 0 to 30) were consistent with generally positive beliefs and attitudes about SDM and its appropriateness in clinical practice. Higher scores on SCDA beliefs (range 0-18) were consistent with generally positive beliefs and attitudes about the Statin Choice Decision Aid and its appropriateness and usefulness in clinical practice.

Table 2: Adjusted rate of Clinician self-reported use of the SCDA.

	Round 1	Round 2	Round 3
Level of exposure of SCDA: Percentage of occasional or routine use (95% CI)	33.5 (22.5, 44.5)	66.5 (54.9, 78.1)	69.7 (60.2, 79.3)

Figure 1: Clinician's Beliefs about SDM by knowledge and use of SCDA in System 2

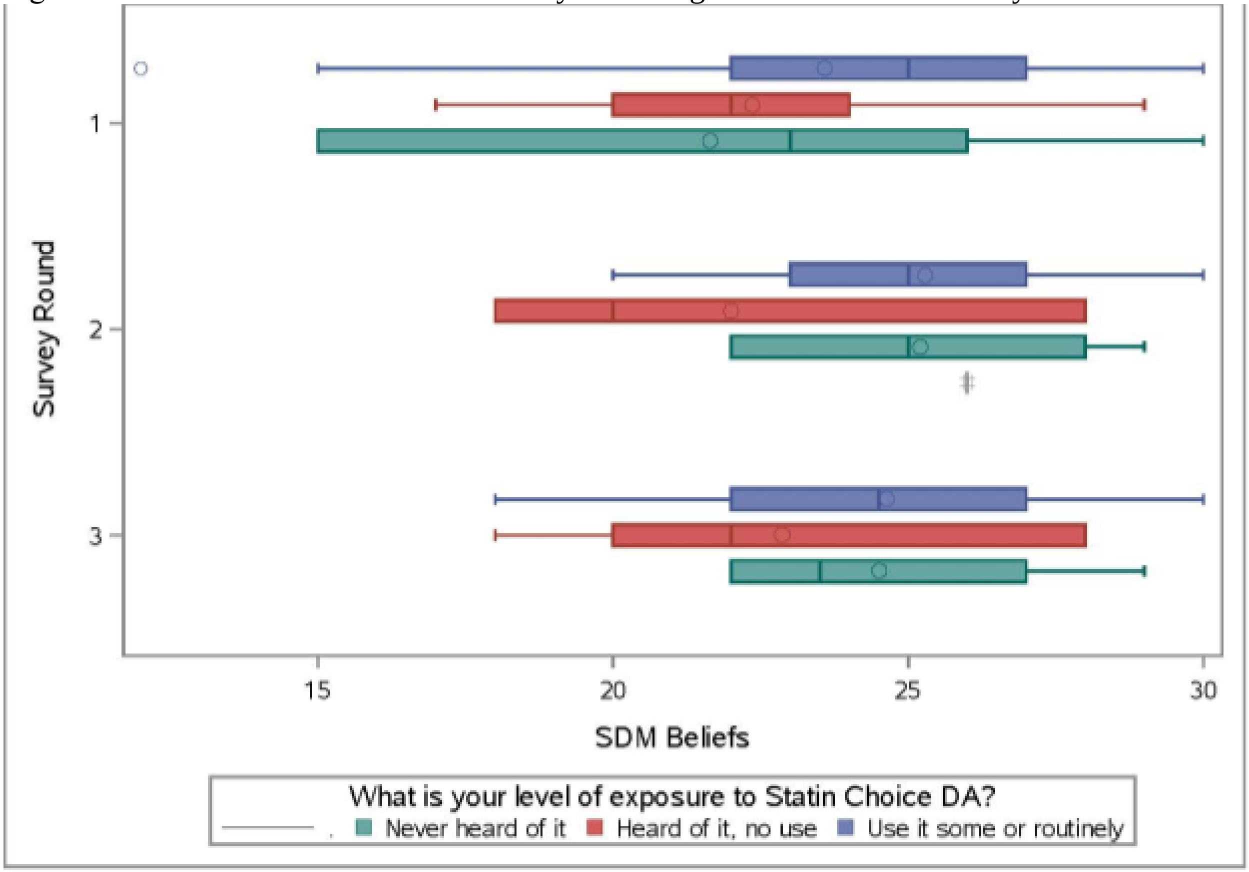

The beliefs of SDM did not differ significantly based off use of SCDA. While on average those who use the tool did have higher beliefs, this impact did not increase over time.

Figure 2: Clinician's Beliefs about SCDA by knowledge and use of SCDA in System 2

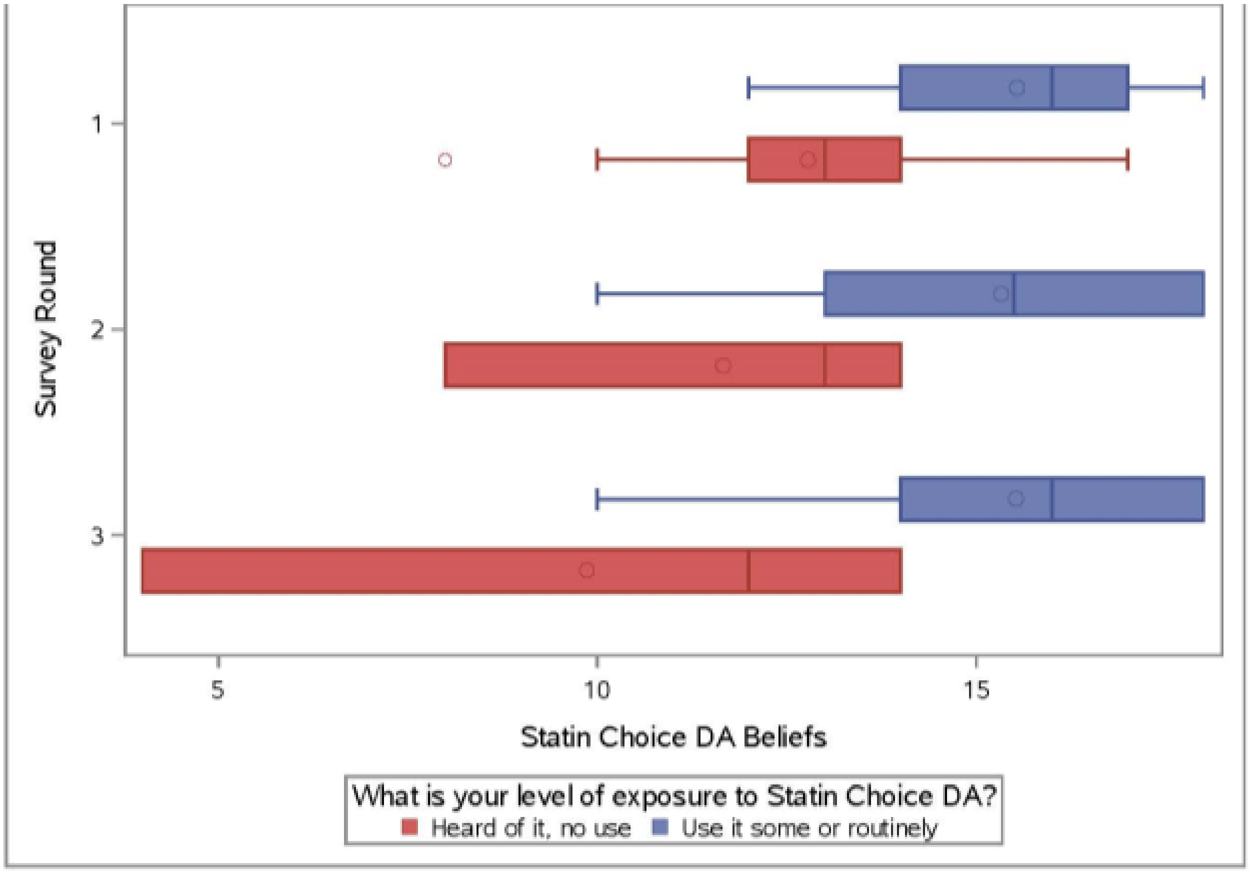

These questions were only asked of clinicians who reported at least hearing of SCDA (Round 1= 31 (72%), Round 2 = 22 (75.9%), Round 3=38 (88.4%)). For SCDA belief the average score did not shift during rounds. For those who did not use the tool, the average belief decreased. This is believed to be a product of those who continue to not use doing so due to the lack of belief in its use.

Figure 3: Web reported usage rate over time in System 2

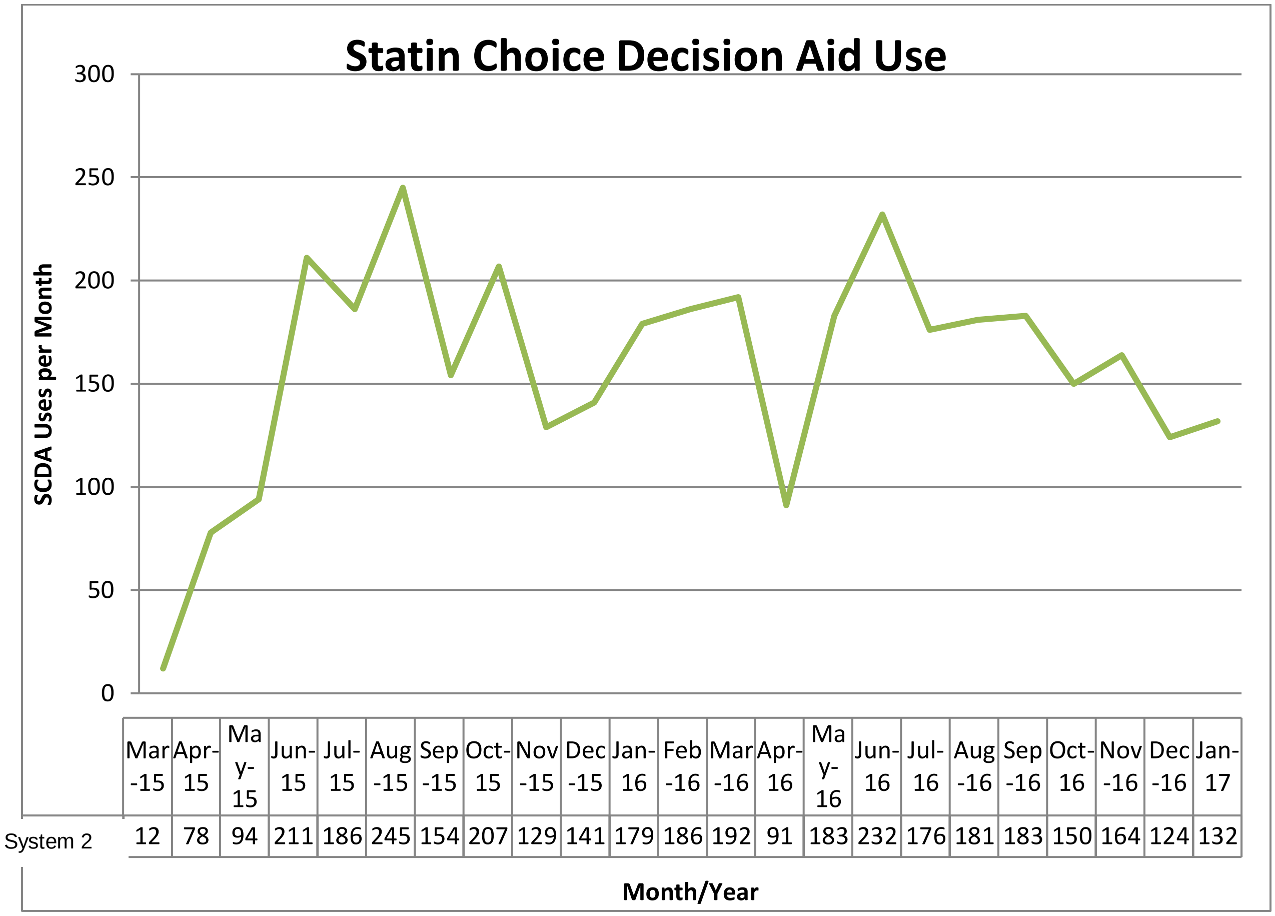

Approximately 3 months into the implementation usage increase to a range that stayed mostly consistent between 150-200 encounters per month with the SCDA being used.

Figure 4: Rate of usage over time of SCDA per clinician in System 2.

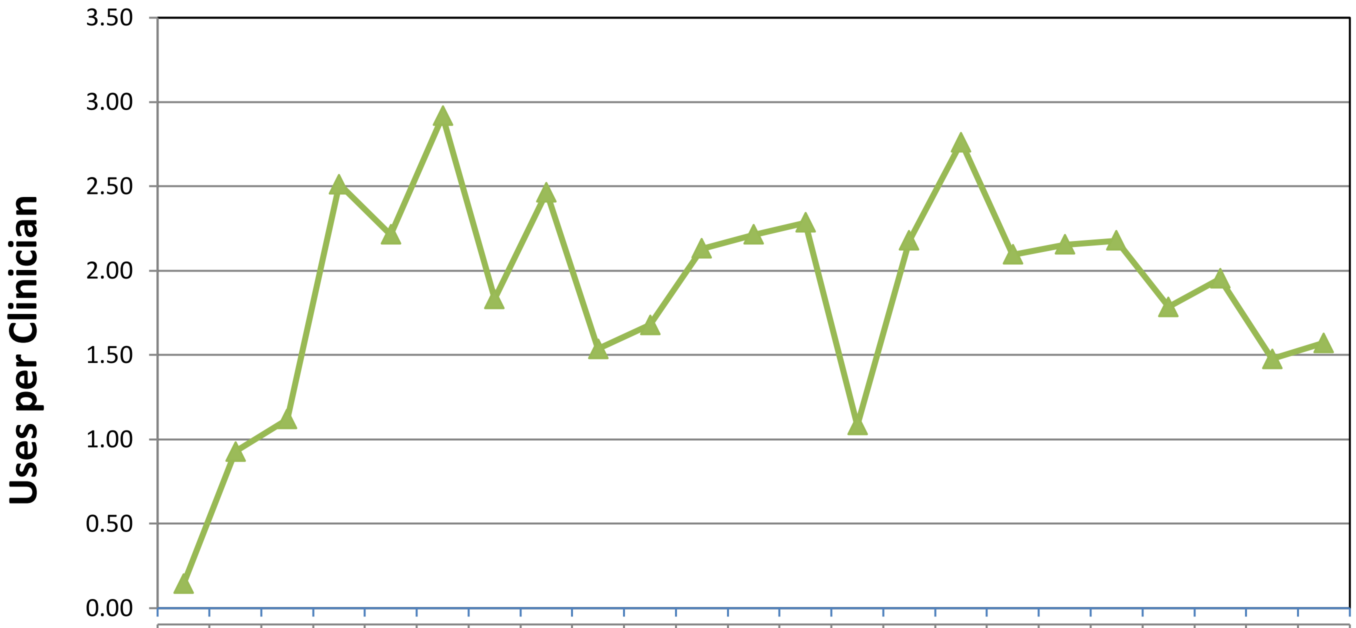

	Mar-15	Apr-15	May-15	Jun-15	Jul-15	Aug-15	Sep-15	Oct-15	Nov-15	Dec-15	Jan-16	Feb-16	Mar-16	Apr-16	May-16	Jun-16	Jul-16	Aug-16	Sep-16	Oct-16	Nov-16	Dec-16	Jan-17
IC	0.1	0.9	1.1	2.5	2.2	2.9	1.8	2.4	1.5	1.6	2.1	2.2	2.2	1.0	2.1	2.7	2.1	2.1	2.1	1.7	1.9	1.4	1.5

Of the 26 clinicians provided to the research team for participation in the research team, 13 clinicians responded to the baseline survey. The clinicians were surveyed at 3 time points: Baseline (Round 1), 6 months post baseline (Round 2) and 12 months post baseline (Round 3).

Table 1: System 3 Clinician Demographics from Round 1

	System 3 (N=13)
**What is your degree within your health care profession?**	
MD/DO	10 (76.9%)
PA	0 (0.0%)
NP	3 (23.1%)
Other	0 (0.0%)
**What is your gender?**	
Female	7 (53.8%)
Male	6 (46.2%)
**What is your age?-Years:**	
Mean (SD)	44.1 (13.8)
Median	41.0
Q1, Q3	34.0, 54.0
Range	(26.0-66.0)
**Race**	
White	13 (100.0%)
**Are you of Hispanic or Latino origin or descent?**	
Yes	0 (0.0%)
No	13 (100.0%)

Response rates for clinicians:

Responded to all rounds	Round 1 & 3	Round 2 & 3	Round 3 only
10 (38.5%)	3 (11.5%)	3 (11.5%)	1 (3.8%)

Response rate by Round for clinicians:

Round 1	Round 2	Round 3
13 (50%)	13 (50%)	17 (65.4%)

Clinicians were asked on their knowledge of and use of the statin choice decision aid as well as a series of questions that assessed their beliefs on shared decision making (SDM Beliefs) and the Statin Choice Decision Aid (SCDA Beliefs). Higher scores on SDM beliefs (range 0 to 30) were consistent with generally positive beliefs and attitudes about SDM and its appropriateness in clinical practice. Higher scores on SCDA beliefs (range 0-18) were consistent with generally positive beliefs and attitudes about the Statin Choice Decision Aid and its appropriateness and usefulness in clinical practice.

Table 2: Adjusted rate of Clinician self-reported use of the SCDA.

	Round 1	Round 2	Round 3
Level of exposure of SCDA: Percentage of occasional or routine use (95% CI)	73.1 (58.8, 87.5)	92.2 (82.4, 100)	93.8 (86.4, 100)

Figure 1: Clinician's Beliefs about SDM by knowledge and use of SCDA in System 3

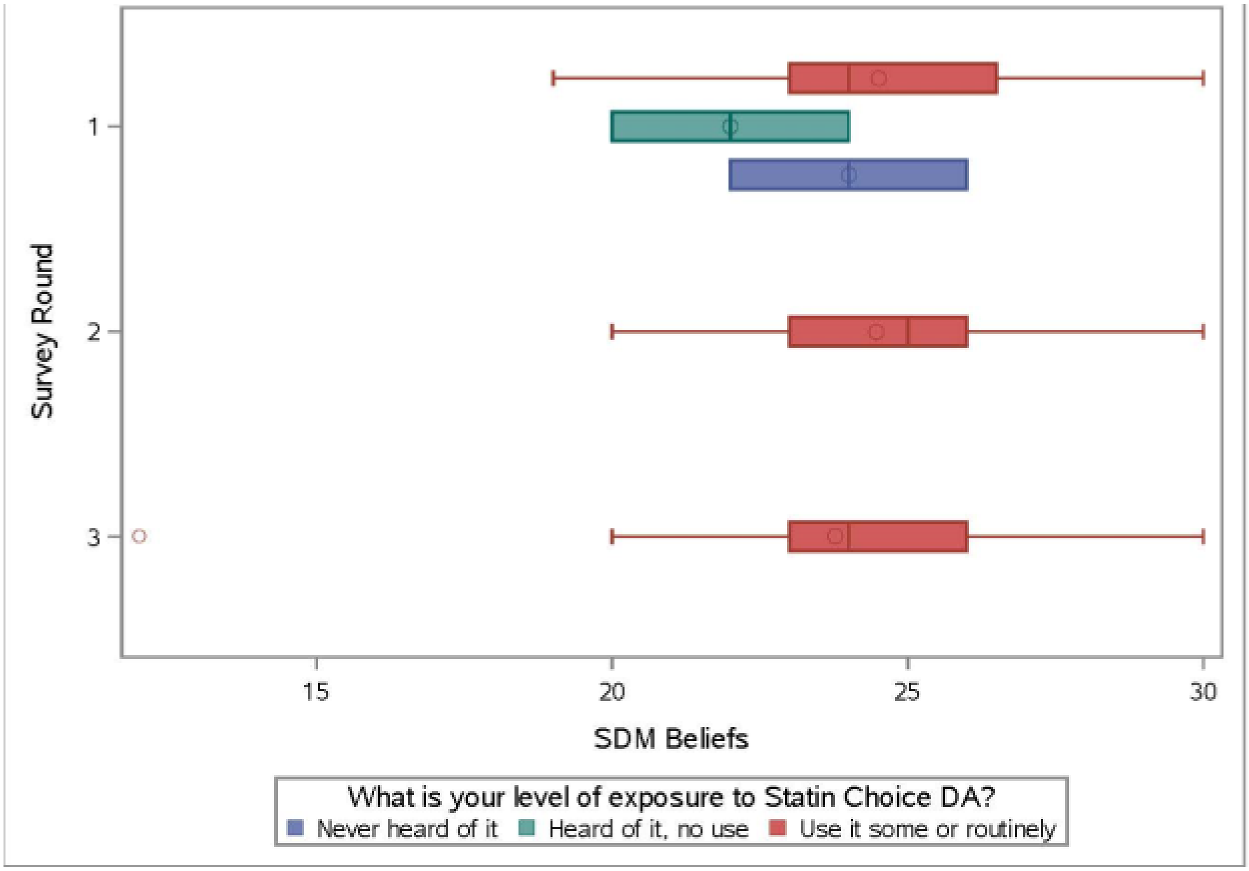

By Round 2, all clinicians that responded to the surveys had awareness of the tool and had used it either occasionally or routinely. The average SDM beliefs did not significantly differ by over time; there was a shift in Round 2 where the average was higher than seen in either of the other rounds.

Figure 2: Clinician's Beliefs about SCDA by knowledge and use of SCDA in System 3

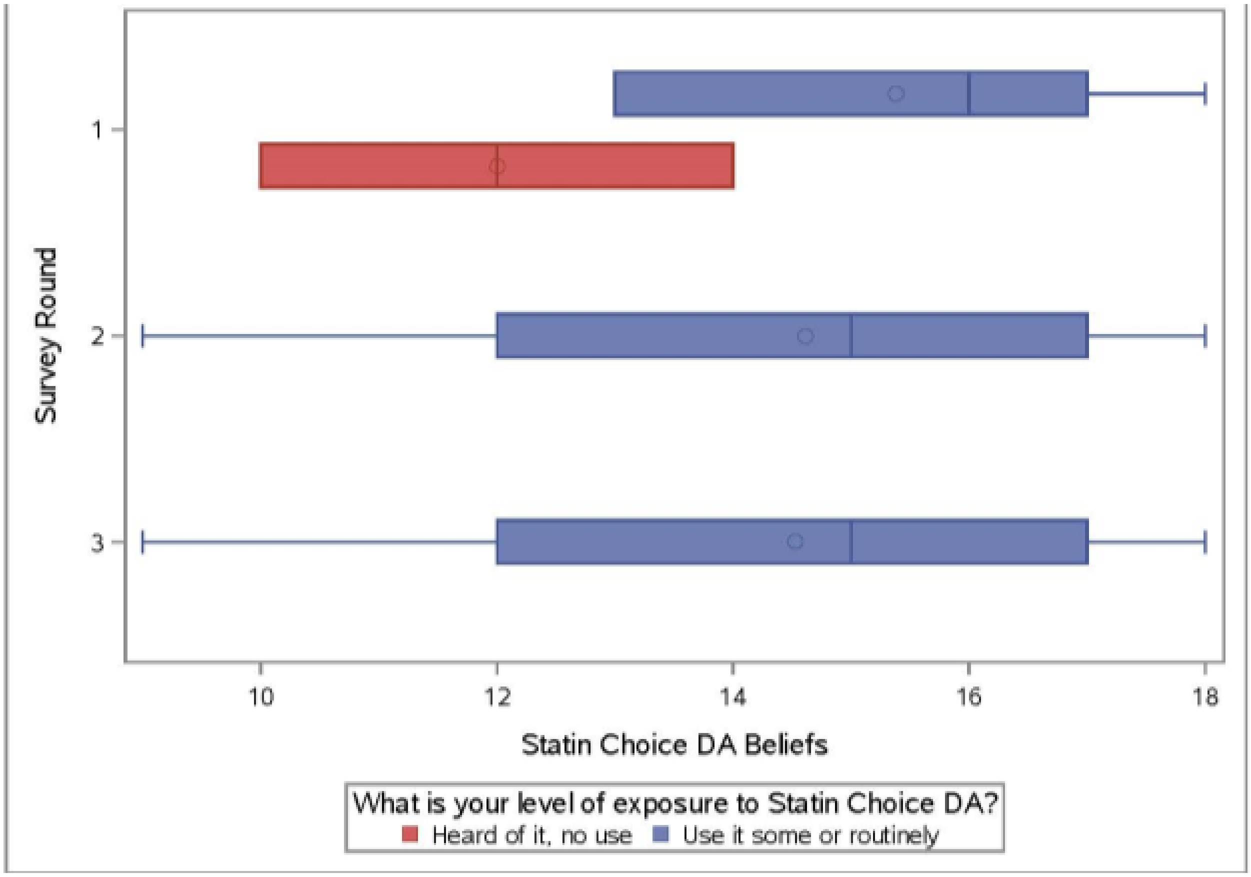

These questions were only asked of clinicians who reported at least hearing of SCDA (Round 1= 10 (77%), Round 2 = 13 (100%), Round 3=17 (100%)). For SCDA belief the average score decreased from Round 1 to Round 2 for those whom use it, there was no change from Round 2 to Round 3. No statistical difference was found.

Figure 3: Web reported usage rate over time in System 3

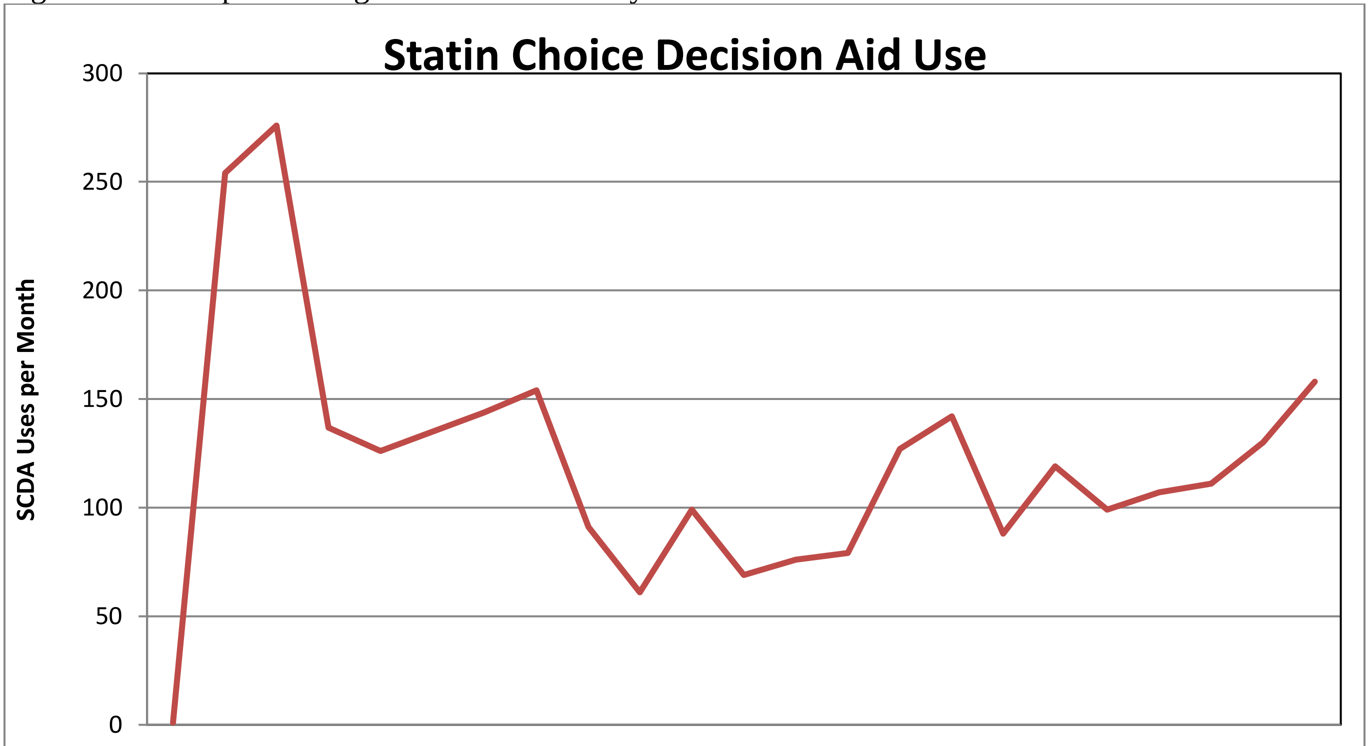

	Mar-15	Apr-15	May-15	Jun-15	Jul-15	Aug-15	Sep-15	Oct-15	Nov-15	Dec-15	Jan-16	Feb-16	Mar-16	Apr-16	May-16	Jun-16	Jul-16	Aug-16	Sep-16	Oct-16	Nov-16	Dec-16	Jan-17
System 3	1	254	276	137	126	135	144	154	91	61	99	69	76	79	127	142	88	119	99	107	111	130	158

While the first two full months of implementation saw the highest usage of the tool, the later months saw consistently high rates of use.

Figure 4: Rate of usage over time of SCDA per clinician in System 3.

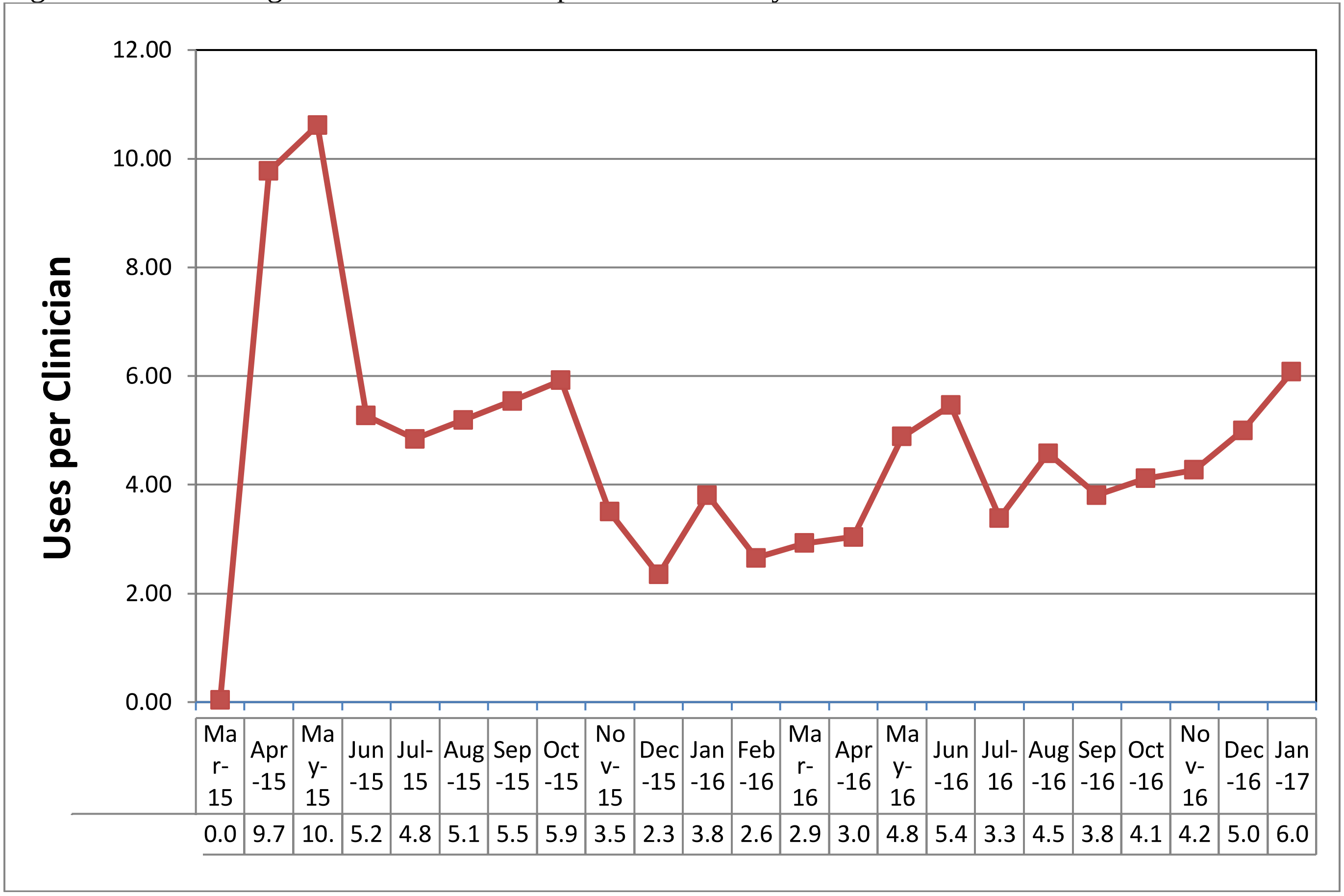

Supplement: Supplementary file 2 — Quantitative and Qualitative Data Summaries. (PDF 1866 kb) [file 12913_2019_4055_MOESM2_ESM.pdf]
